# The Footprint of Kynurenine Pathway in Neurodegeneration: Janus-Faced Role in Parkinson’s Disorder and Therapeutic Implications

**DOI:** 10.3390/ijms22136737

**Published:** 2021-06-23

**Authors:** Tapan Behl, Ishnoor Kaur, Aayush Sehgal, Sukhbir Singh, Saurabh Bhatia, Ahmed Al-Harrasi, Gokhan Zengin, Adrian Gheorghe Bumbu, Felicia Liana Andronie-Cioara, Aurelia Cristina Nechifor, Daniela Gitea, Alexa Florina Bungau, Mirela Marioara Toma, Simona Gabriela Bungau

**Affiliations:** 1 Department of Pharmacology, Chitkara College of Pharmacy, Chitkara University, Punjab 140401, India; ishnoorkaur7@gmail.com (I.K.); aayushsehgal00@gmail.com (A.S.); sukhbir.singh@chitkara.edu.in (S.S.); 2 Amity Institute of Pharmacy, Amity University, Gurugram, Haryana 122412, India; sbsaurabhbhatia@gmail.com; 3 Natural and Medical Sciences Research Centre, University of Nizwa, P.O. Box 33, PC 616 Birkat Al Mouz, Nizwa 611, Oman; aharrasi@unizwa.edu.om; 4 Department of Biology, Faculty of Science, Selcuk University Campus, Konya 42130, Turkey; biyologzengin@gmail.com; 5 Department of Surgical Disciplines, Faculty of Medicine and Pharmacy, University of Oradea, 410073 Oradea, Romania; abumbu@uoradea.ro; 6 Department of Psycho-Neuroscience and Recovery, Faculty of Medicine and Pharmacy, University of Oradea, 410073 Oradea, Romania; felicia_cioara@yahoo.com; 7 Analytical Chemistry and Environmental Engineering Department, Polytechnic University of Bucharest, 011061 Bucharest, Romania; aureliacristinanechifor@gmail.com; 8 Department of Pharmacy, Faculty of Medicine and Pharmacy, University of Oradea, 410028 Oradea, Romania; gitea_daniela@yahoo.co.uk (D.G.); mire.toma@yahoo.com (M.M.T.); 9 Faculty of Medicine and Pharmacy, University of Oradea, 410073 Oradea, Romania; pradaalexaflorina@gmail.com; 10 Doctoral School of Biomedical Sciences, University of Oradea, 410087 Oradea, Romania

**Keywords:** Parkinson’s disease, Janus-faced role, kynurenine pathway, neurotoxic, quinolinic acid, neuroprotective, kynurenic acid

## Abstract

Progressive degeneration of neurons and aggravation of dopaminergic neurons in the substantia nigra pars compacta results in the loss of dopamine in the brain of Parkinson’s disease (PD) patients. Numerous therapies, exhibiting transient efficacy have been developed; however, they are mostly accompanied by side effects and limited reliability, therefore instigating the need to develop novel optimistic treatment targets. Significant therapeutic targets have been identified, namely: chaperones, protein Abelson, glucocerebrosidase-1, calcium, neuromelanin, ubiquitin-proteasome system, neuroinflammation, mitochondrial dysfunction, and the kynurenine pathway (KP). The role of KP and its metabolites and enzymes in PD, namely quinolinic acid (QUIN), kynurenic acid (KYNA), 3-hydroxykynurenine (3-HK), 3-hydroxyanthranillic acid (3-HAA), kunurenine-3-monooxygenase (KMO), etc. has been reported. The neurotoxic QUIN, N-methyl-D-aspartate (NMDA) receptor agonist, and neuroprotective KYNA—which antagonizes QUIN actions—primarily justify the Janus-faced role of KP in PD. Moreover, KP has been reported to play a biomarker role in PD detection. Therefore, the authors detail the neurotoxic, neuroprotective, and immunomodulatory neuroactive components, alongside the upstream and downstream metabolic pathways of KP, forming a basis for a therapeutic paradigm of the disease while recognizing KP as a potential biomarker in PD, thus facilitating the development of a suitable target in PD management.

## 1. Introduction

The greatest obstacle associated with neurodegenerative disorders is that they are incurable, and the deterioration is progressive with time and age. These pathologies vary in symptoms, pathological features, and drug candidates. Parkinson’s disease (PD), as well as Alzheimer’s disease (AD), are considered to be the most prevalent among the neurodegeneration-related disorders [1]. Recently, PD was reported to affect about 6.1 million people, in comparison to 2.5 million people in 1990 [2]. Accounting for the 21.7% of age standardized disease prevalence rate, the disease progressively accelerated during all these years. In 2016, PD led to 211,296 deaths around the globe [3]. The symptoms in PD include tremors, rigidity, bradykinesia, and disrupted posture, parallel to retardation in mental processing, speech problems, memory losses, and leaning inability.

The prime pathological features of the disease include loss of dopaminergic neurons in the substantia nigra pars compacta (SNpc) and alleviated dopamine (DA) levels in the striatum [4,5]; however, the specific cause of the disease is still to be investigated. When the loss of dopaminergic neurons reaches 70% to 80%, it is marked with the appearance of associated signs and symptoms [2]. Therefore, this period (from the beginning of the loss of dopaminergic neurons in the brain to the identification of clinical signs and symptoms) marks the basis for development of effective treatment therapies for the disease. Despite the availability of numerous treatment options, no therapy is completely effective for this disorder. The therapies available for the disease are associated with hindrances, such as inability to cross the blood–brain barrier (BBB), side effects, limited life span, etc., alongside primary challenges such as the development of sensitive and reliable of biomarkers for disease detection [6,7]. Thus, there is a dire need to develop novel treatment options for PD, with greater specificity and selectivity for the disease targets and limited side effects.

Various metabolomics studies have been performed to identify specific targets and biomarkers in PD pathogenesis. The metabolism of tryptophan (TRP) in association with the kynurenine pathway (KP) has been employed in multiple neurodegenerative and psychiatric diseases, cancer, inflammation, obesity, and diabetes [8]. The metabolites linked to KP are responsible for exerting physiological actions by interacting with intracellular or extracellular receptors in the peripheral and central nervous system (CNS) regions [8]. The activity at α7 nicotinic acetylcholine receptors and glutamate receptors is regulated by KP metabolites, which in turn influences the excitatory neurotransmission [9,10] and can further affect the immune-mediated responses, by acting at the transcription factor aryl hydrocarbon receptor (AhR) [10,11]. An important cofactor, nicotinamide adenine dinucleotide (NAD+), associated with energy metabolism, is produced as an end product of KP [12,13]. Large neutral amino acids transporters and enzymes modulate the concentration of KP metabolites in multiple tissues, where its actions are controlled by inflammation and exercise, along with microbiota composition in the gut [8].

The enzymes associated with KP have been recognized as reliable and promising target candidates for treatment of neurodegenerative diseases. Ageing and inflammation can alter the KP metabolism and availability of TRP [14], thus elevating the susceptibility towards age-dependent neurological disorders, including PD [15,16]. Chronic inflammation of the intestine, gut microbiota changes, and α-synuclein aggregation are the pivotal features of PD pathogenesis [17].

The review focuses on the role of KP in PD excitotoxicity, where the KP metabolites were found to play a dual role in disease progression. Quinolinic acid (QUIN) was identified as the neurotoxic metabolite, whereas, kynurenic acid (KYNA) was identified as a neuroprotective metabolite in the brain [8]; they have been found to exhibit antagonistic actions in KP, thereby justifying the Janus-faced role of KP in mitigation of the disease. Moreover, the literature also elaborates other features of impaired KP metabolism, such as elevated KYN/TRP ratio and enhanced 3-HK/KYNA ratio, in the CNS, alongside elevated serum levels of KYN/TRP ratio, which have been observed in the periphery, however, they are very limited [8]. Moreover, the authors aim to highlight the role of KP in PD, while elaborating the antagonizing actions of its neurotoxic and neuroprotective metabolites, QUIN and KYNA. These can uncover the possibility of development of a novel therapeutic regime in PD, thereby giving an opportunity to researchers all over the world to investigate the possible involvement of KP metabolites and enzymes in PD progression and establish its significance as a potential target. The manuscript details the role of upstream and downstream KP metabolites in PD, followed by identifying KP as a biomarker in disease detection, and finally elaborating the therapeutic considerations related to it.

## 2. The Kynurenine Pathway

The KP functions as a significant target for PD, which is the major focus area of this review. This TRP metabolism pathway aids in the generation of NAD+. QUIN and KYNA are the two significant metabolites formed in the pathway, where any differences in these metabolites results in PD development, as discussed further in the review. As a primary degradation pathway of essential amino acid, TRP, the KP results in the formation of NAD+, an essential co-factor [18], aiding as well as in immune response regulation.

KP metabolism affects the processes associated with NAD+, by directly regulating the production of NAD+, which further modulates the tri-carboxylic acid cycle and function of the mitochondria [19]. The NAD+ metabolism and function of mitochondria can be affected by KP by regulating ROS concentration. The KP and NAD+ are divided to microglial cells and astrocytes in the CNS, with only a few KP metabolites, such as KYN, TRP, and 3-HK, able to cross the BBB [19]. The different expressions of IDO-1, IDO-2, and TDO2 in the tissue govern the entry of TRP into KP [20]. QUIN, the major precursor of NAD+ in KP, is not contained in the liver, even post TRP loading, which depicts NAD+ processing in this tissue [21]. After accumulating in activated immune cells, QUIN might function as a reservoir for production of NAD+, further providing a substrate for the activity of PARP, which is required to fight the DNA damage done by elevated oxidative stress during immune activity. As an alternative, the cells of the immune system may eliminate QUIN, and use its pro-oxidant features to attack the invading pathogens [19].

The immune tolerance, in association with pregnancy and severity of tumors, is developed by induction of indoleamine-2,3-dioxygenase (IOD-1), which regulates activation of KP and mitigation of TRP [22,23]. IDO-1 is a rate limiting enzyme of KP, which is the primary pathway of TRP catabolism. IDO-1 has been found to be involved in immune tolerance and contributes to the maintenance of T cell homeostasis of self-antigen tolerance during the inflammatory conditions [24]. Its neuronal functions have initially targeted its involvement in brain tumors and autoimmune diseases. The ratio of KYN to TRP is used to assess the activity of IDO-1 because of the ability of IDO-1 to convert TRP to KYN [24]. The concentration levels of KYN and TRP in the serum and CSF of 22 patients with PD was compared with 11 control subjects, where the ratio of KYN to TRP was found to be elevated in both the serum and CSF of patients with PD [25]. In addition to this, the serum and CSF ratio of KYN to TRP correlates with concentration of neopterin, depicting activation of immune system and is also associated with the severity of the disease [24].

The clonal expansion of T cells is completely hindered by activation of IDO-1 in the dendritic cells [26]. The three theories proposed are: TRP degradation-mediated suppression of T cells proliferation by ameliorating the supply of this important amino acid; suppression of some immune cells by certain downward KP metabolites; and KP metabolites, such as kynurenine (KYN), cinnabarinic acid (CA), and KYNA, promote AhR activation, which facilitates the dendritic cell-mediated production of transforming growth factor beta-1 (TGFβ-1) resulting in development of tolerance towards the disease [27]. The neuronal KP expression is yet to be further explored; however, the enzyme moieties associated with KP are completely expressed in monocytes such as macrophages and microglial cells [28,29]. These enzymes were reported to be only partly present in oligodendrocytes, neurons, endothelial cells, and astrocytes in humans.

The astrocytes and neuronal cells are considered to be neuroprotective, whereas the infiltrating macrophages and activated microglia are considered to be neurotoxic [30]. Therefore, multiple products of KP can have neuroprotective, neurotoxic, and immunomodulatory effects [31]. QUIN, an excitotoxin, is reported to be the most significant among them, which results in the death of neurons [32,33]. Only infiltrating macrophages and activated microglia, and not astrocytes and neurons, are responsible for the production of QUIN in the brain [28]. The astrocytes produce KYNA, which antagonizes the effects of ionotropic glutamate receptors resulting in hindering the actions of QUIN as well as excitotoxins. However, in diseased conditions, the production of QUIN is in excess, unlike KYNA, which fails to sufficiently block QUIN [10]. The two KP metabolites exhibit a sharply contrasting aspect of characteristics; therefore, they are depicted to play a Janus-faced role in PD.

Another intermediate metabolite of KP is 3-hydroxykynurenine (3-HK), which exerts neurotoxic actions and produces free radicals at nanomolar concentrations, resulting in neuronal degeneration and apoptosis, and also causes elevated metal toxicity in rat astrocyte culture [34,35]. Additionally, hydrogen peroxide and superoxide ions are produced by 3-HK, which results in induction of copper-dependent damage to oxidative proteins [35,36]. The neurotoxic effects of QUIN can be effectively synergized by 3-HK [10]. The human primary neurons produce an endogenous metal chelator, picolinic acid in micromolar concentrations, which exerts neuroprotective actions [30,37]. It is noteworthy, that KP results in the generation of KYNA or NAD+ or picolinic acid in physiological states and under inflammatory conditions, QUIN is over-expressed along with other pro-inflammatory and neurotoxic moieties [38].

**Figure 1:**
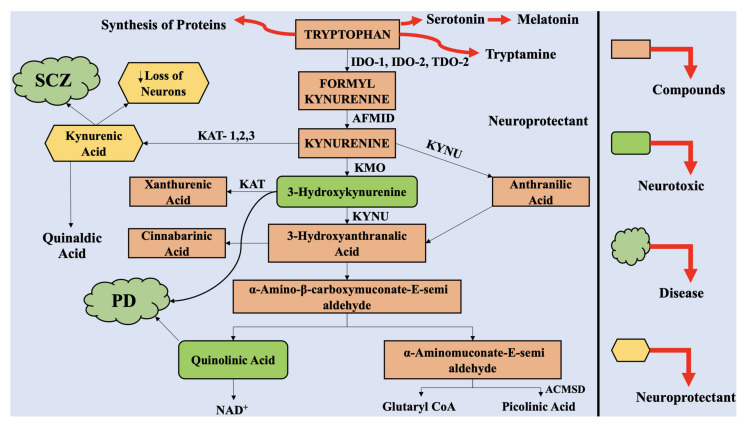
An overview of multiple steps involved in the kynurenine pathway, forming two significant metabolites: quinolinic acid and kynurenic acid. Legend: SCZ—schizophrenia, KYNU—kynureninase, IDO—indoleamine 2,3-dioxygenase, AFMID—arylformamidase, KMO—kynurenine 3-monooxygenase, KAT—kynurenine aminotransferase, PD—Parkinson’s disease, ACMSD—2-amino- 3-carboxymuconate-6-semialdehyde decarboxylase, CoA—coenzyme A, NAD+—nicotinamide adenine dinucleotide, TDO—tryptophan-2,3-dioxygenase.

For many years, many investigations have been targeting the initial steps in KP, comprising of TRP transformation to KYNA, corresponding to activity of IDO-1 [10]. Over the last ten years, the significance of the middle and downstream steps of KP has risen to higher levels. The intermediate metabolites of KP have been reported to play a significant role in neurotoxicity, as per multiple studies, such as immunomodulatory role of 3-HK as well as neurodegeneration inducted by CA and 3-hydroxyanthraanilic acid (3-HANA) [30]. Figure 1 provides a layout of the KP and its different steps.

## 3. The Interaction of Kynurenine Pathway with the Central Nervous System

The activity of IDO/TDO regulates the initial rate-determining step of KP, alongside enzymes that exhibit varying affinities for specific isoforms of TRP, slow sequence similarity, and are modulated by different mechanisms [39,40]. Tryptophan-2,3-dioxygenase (TDO) is a deoxygenase, which contains heme encoded by TDO-2 gene [41,42]. The L-TRP is oxidized as a result of TDO heme group reduction, forming heme-Fe^2+^ from heme-Fe^3+^, thus the activity of L-TRP is found to be regulated by reactive oxygen species (ROS) and reducing agents [43]. Furthermore, proinflammatory cytokines may further promote activation of TDO. However, this is expected to happen indirectly via glucocorticoid receptor activation. The immune response promotes the release of proinflammatory cytokines, which are responsible for directly inducing IDO-1 [44].

One of the prime regulators of IDO-1 transcription is interferon-gamma (INF-γ), which binds to one out of two INF-γ activation sites (GAS) on the 5′ flanking site of IDO-1 gene [45]. Therefore, INF-γ-dependent activation of IDO-1 can be promoted by other proinflammatory cytokines, such as interleukin-1 beta (IL-1β) and Toll-like receptor (TLR) agonists, which synergize the activity of IDO-1 as a response to the proinflammatory stimuli [46]. It is noteworthy that the L-KYN/TRP ratio is elevated by IDO-1 activation [47,48]. Additionally, the proinflammatory stimuli can also affect the activity of kynurenine 3-monooxygenase (KMO), which are elevated in the hippocampus and whole brain extracts as a result of systemic administration of LPS in rats [49,50,51,52].

On the other hand, in gene transcription and coding, kynureninase (KYNU) and KMO both are promoted by IL-1β treatment in the human hippocampal progenitor cells [48]. Microglial activation is also mediated by immune response, along with influx of macrophages and proinflammatory cytokines in the brain. The cytokine influx or macrophages have the tendency to cause potential alterations to the concentration and ratio of KP metabolites in the CNS, on account of approximately 20-fold greater capacity of macrophages for QUIN production as compared to microglia [28,53]. The concentrations of TDO-2 and IDO-1 are much lesser in the brain, unlike the periphery, despite the capability of TRP to cross the BBB [54]. Therefore, the BBB permeable, L-KYN, which is peripherally produced and taken up by the glial cells [55,56], is responsible for initiation of KP metabolism (about 60%) in the neuronal tissue. The two primary arms associated with metabolism of L-KYN are detached physically in the brain, where KMO is not expressed in astrocytes, but in the microglia; therefore the 3-HK arm of KP resulting in the production of QUIN occurs in the microglia [53]. On the other hand, KYN aminotransferases (KATs) are not found in microglia but are contained in the astrocytes; therefore, KYNA production in the CNS takes place in the astrocytes [57,58].

Numerous factors, such as concentration of glutamate receptor agonists, potassium ion levels, and concentration of glucose, affect the synthesis and release of KYNA from astrocytes [59]. D-amphetamine, which inhibits reuptake of DA, ameliorates the concentration of KYNA in the brain, without decreasing its levels in the periphery, consequently implying the regulatory function of DA. On the other hand, L-KYN levels are not affected [60]. Furthermore, it has been found that the neuronal signaling is essential for KYNA regulation, on account of loss of further influences in the absence of neuronal cells. The abundance of 3-HANA and 3-HK affects the concentration levels of QUIN [61]. Similarly, the actions of enzymes KYNU, 3-HANA 3,4-dioxygenase (3-HAO), and KMO also affect QUIN levels [62,63]. For the use of QUIN and KYNA in the CNS, these metabolites must be produced locally, as both are deprived of effective active transport processes and lack the ability to penetrate BBB. Changes in the blood levels of periphery- produced 3-HK can affect the relative amounts of QUIN with respect to KYNA in the CNS, as 3-HK is able to cross the BBB [64].

Numerous KP enzymes have significant neuroprotective, neurotoxic, and immunomodulatory roles [15]. Maximum number of KP metabolites produce 3-HK in the astrocytes and microglia of the brain [57]. Neurodegeneration and neuronal apoptosis is induced by 3-HK in healthy cells by production of free radicals [34,65]. On the other hand, in the diseased cells, 3-HK is transformed into QUIN, which exhibits significant role in dysfunction of neurons, inducing neurotoxicity [66]. However, the KYNA metabolite is responsible for inhibiting the actions of QUIN and excitotoxins [57,65]. The glutamatergic signaling is altered by the ratio of KYN metabolites, which provide protection against excitotoxicity, induced by N-methyl-D-aspartate (NMDA) receptors, which suggests the prime role of KP in physiological states of the body [8]. Only IDO-1 is responsible for KP activation during neuroinflammation, which could be acute, low-grade, or severely progressive. IDO-1 is known to be activated by multiple inflammatory mediators, such as TNF-α, INF-γ, lipopolysaccharides, TLRs-1,6,9, and viral and amyloid proteins [10].

The IDO-1, IDO-2, and KMO enzymes, along with the KYN metabolites, have also been reported to contribute to neuropathic pain, as well as migraine, headache, depression etc. [67]. The association of numerous enzymes of KP, such as KMO, IDO-1, IDO-2, TDO, KYNU etc., with neuropathic pain has been investigated in a rat model of chronic constriction injury (CCI) at the spinal cord as well as the levels of dorsal root ganglia [68].

The levels of these enzymes were assessed in the microglial cells, stimulated by LPS, and recovered from cerebral cortices, which reported that all enzymes, other than TDO, were derived from this cell population. Enhanced mRNA concentration of IDO-2, KMO, and HAOO were reported at the spinal cord level after seven days of CCI without any alterations in the TDO mRNA levels. These events took place parallel to the microglial/macrophage activation [67]. Intraperitoneal injection of minocycline was repeatedly administered both before and after CCI, which mitigated the concentration of IDO-1 and KMO enzymes, alongside ameliorated thermal and tactile hypersensitivity. Moreover, inhibitors of KMO and IDO-2, UPF-648 and 1-methyl-D-tryptophan (1-D-MT), mitigated hypersensitivity to thermal and mechanical stimuli. Thus, this evidently supports the role of IDO and KMO in the pathogenesis of neuropathic pain [67].

As a prime catabolic enzyme of TRP in the neuronal tissue, IDO-1 is significant regulator of KP [10]. The serum and CSF samples of PD patients were reported to exhibit enhanced ration of KYN to TRP, as the result of elevated IDO and TDO levels. The PD population has been recognized to constitute elevated number of single nucleotide polymorphisms (SNPs); however, it is still unclear if the IDO-associated genetic disturbances lead to KYN metabolism impairment and PD [69]. Allelic discrimination assay was used to perform SNP analysis of IDO1, along with fluorescently labelled TaqMan probes. Further, a subgroup analysis was carried out depending upon the onset of the disease in patients. The three variants of 105 PD patients with no comorbidities: intronic variant rs7820268, frame shifts variant rs34155785, and promoter region variant rs9657182, were evaluated and compared to 129 healthy control subjects. However, the A alleles of rs7820268 SNP or rs9657182 SNP carriers were found to aid the late onset of disease in PD patients, as compared to non-carriers, as reported by the subgroup analysis [69]. This investigation revealed the impact of SNPs of IDO1 on the age onset of PD and the function of SNP genotypes as a risk biomarker of PD [69].

The physiological concentration of QUIN in the cerebrospinal fluid (CSF) and brain is approximately 100 nM, and it exhibits hormesis action. QUIN treatment results in elevation of NAD+ production, within the human neuronal cells, alongside enhanced proliferation of stem cells [70]. However, the concentration of QUIN is increased to 1200 nM in disease conditions, which can lead to acute, chronic, or progressively severe functions of neurons or death of neuronal cells by a minimum of nine varying processes [71]. At pathological concentrations, between 150 to 1200 nM, QUIN functions as NMDA receptor agonists. Previously, two studies reported the selective actions of QUIN on NMDA receptor, along with differences in actions, mediated by QUIN on NMDA, at particular neuronal areas [72]. Vandresen-Filho et al. confirmed the above statement and reported that the hippocampus, cerebral cortex, cerebellum, and striatum have varying susceptibility towards QUIN induced oxidative stress [73]. The neuronal cells contained in the striatum, hippocampus, and neocortex, exhibit sensitivity towards QUIN; however, the neurons present in the spinal cord and cerebellar region are comparatively less sensitive. These differences are associated with alterations in the configurations of the NMDA receptor [74].

It was depicted that the motor neuron could be completely protected from QUIN-mediated excitotoxicity by using NMDA receptor (NMDAR) antagonist combinations [75]. QUIN has the ability to cause elevated micro-environment glutamate levels and neurotoxicity by enhancing the release of glutamate by neuronal cells, blocking its astrocyte-mediated uptake and inhibiting astroglial glutamine synthetase enzyme [76].

Furthermore, QUIN has the tendency to promote indirect peroxidation of lipids [77,78]. ROS are formed by electron transfer from QUIN-iron complex to oxygen, which further promotes peroxidation of lipids [79]. QUIN can potentiate its own toxicity profile, as well as of the other excitotoxins, in association with energy depletion. The other excitotoxins, derived from the immune system, are synergized by toxicity mediated QUIN. Additionally, the integrity of the BBB is disrupted by the actions mediated by QUIN [80]. Moreover, certain regions of the striatum and hippocampus are reported to exhibit greater sensitivity towards QUIN mediated excitotoxic actions, as compared to others [81].

Oxidative stress is induced in the astrocytes and neuronal cells by QUIN, which results in the death of the glial cells and neurons, by energy restriction. The phosphorylation of cellular structural proteins, such as glial fibrillary acidic protein (GFAP) in astrocytes and neurofilament in neuronal cells are elevated by QUIN, resulting in the destabilization of the cytoskeleton [82,83]. It has been reported that the tau phosphorylation in neuronal cells in humans is elevated by QUIN, which further co-localizes with hyperphosphorylated tau in neuronal cells in cortex of AD-affected neuronal tissue [83].

The cellular agents, which provide support to the neuronal cells, i.e., astrocytes, are also altered by QUIN, which also dysregulated astroglial functions and promote death of the glial cells [84]. As a result, dysregulated function and death of neuronal cells take place [10]. Furthermore, QUIN exerts proinflammatory effect on astrocytes, followed by generation of proinflammatory cytokines as well as chemokines, such as monocyte chemotactic protein 1 (MCP-1), IL-1β, and IL-8 in astrocytes [76]. The activity of glutamine synthetase is retarded by QUIN in a dose dependent manner in the astrocytes in humans, which leads to disruption of glutamine/glutamate cycle [76].

Excessive NMDAR stimulation is responsible for regulation of the QUIN gliotoxicity mechanism [84]. It was demonstrated that QUIN is also responsible for killing oligodendrocytes [85]. The expression of cathepsin D (which is a lysosomal aspartic protease) is elevated, and that of Beclin-1 (which is a tumor suppressor protein) is alleviated as the result of QUIN treatment of human neurons and astrocytes. Autophagy disturbances indicate an important mechanism for toxicity mediated by QUIN [86].

The stochastic resonance therapy was reported to influence the TRP metabolism in healthy non-smoking subjects, by ameliorating the TRP, KYNA, and KYN levels that might result in the progression of neuropsychiatric disorders, such as PD, AD, Huntington’s disease (HD), etc. [87,88]. A flame retardant in Drosophila PD model, 2,2′,4,4′-Tetrabromodiphenyl ether (BDE-47), when exposed, resulted in retardation of KYNA formation with an elevation in 3-HK levels [89]. The redox homeo-dynamics is maintained by antioxidant actions of KYNA and xanthurenic acid (XA), which results in reduction of neurodegeneration [90].

## 4. Alteration in KP Metabolites, Parallel to Impairment of Mitochondria Functions, Redox Metals and Oxidative Stress in PD

The early 1990s marked the first description of disrupted KP in PD, where the authors reported elevation in KYNA/TRP and RP/KYN ratios in the putamen, pars compacts, and frontal cortex in PD patients, and the concentration levels of 3-HK were increased in the SNpc and putamen [91]. The frontal cortex region of PD patients who were administered with levodopa (L-DOPA), were marked with ameliorated levels of KYNA and KYN. Additionally, the ratio of KYN to 3-HK was retarded in the SNpc and frontal cortex of patients with PD administered with L-DOPA, but not in the putamen. Another group was marked with decreased KYNA levels in the cortical area, cerebellum as well as caudate of PD patients [92]. As a result of retarded levels of endogenously formed KYNA, its potential to retard QUIN or glutamate-induced excitotoxicity, via NMDA receptors, was retarded [93].

**Figure 2:**
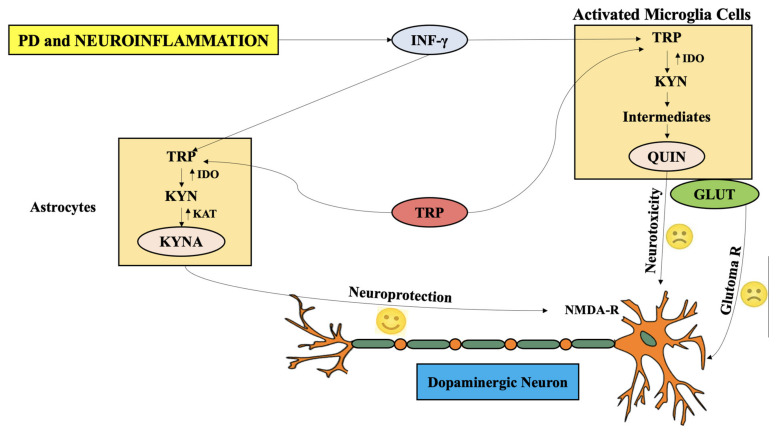
Neuroprotective and neurotoxic effects mediated by KYNA and QUIN in Parkinson’s disorder and neuroinflammation. Legend: TRP—tryptophan, IDO—indoleamine 2,3-dioxygenase, KYN—kynurenine, QUIN—quinolinic acid; NMDAR—NMDA receptor; KAT—kynurenine aminotransferase, GluT—glutamate transporter, KYNA—kynurenic acid, INF-γ—interferon-gamma, **↑** increased expression.

Furthermore, the concentration of KAT-1, the enzyme associated with KP, which aids in KYNA formation, was retarded in the SNpc of mice treated with MPTP toxin [10]. This group also depicted co-expression of TH and KAT-1 in the same neurons of SNpc, and loss of maximum number of nigral neurons as a result of 6-OHDA injected into lateral ventricle of adult rats. Published data also reported expression of KAT-1 in SNpc astrocytes in normal conditions and elevated post 6-hydroxydopamine (6-OHDA) administration, while microglia generated immunoreactive KAT-1 only after administration of 6-OHDA. The serum and CSF biofluids of PD patients was marked with elevated KYN/TRP ratio, as compared to the healthy controls with similar gender and age profiles [94]. The actions of KAT-1 and KAT-2 were predominantly retarded, as evident from the data obtained from post-mortem neuronal PD tissue and mice treated with MPTP, parallel to alleviated levels of KYNA in plasma [95]. The KP has also been marked with alterations in the peripheral organs in PD patients. These investigations provide evidence-based data for catabolic shift of TRP towards QUIN and 3-HK, resulting in retarded concentrations of KYNA, leading to neurotoxic effects and cell death (Figure 2).

As well, the RBCs in the periphery have been reported to exhibit enhanced catalytic activity of KAT-2, which aids in the neuronal production of 75% of KYNA. However, plasma of PD patients is not reported to exhibit any such actions [95]. Increased concentration of KYNA is associated with elevated activity of KAT-2. This action may be associated with the 3-HK release from CNS. Neuronal transportation of KYNA may take place from the periphery by large neutral amino acid carriers to exhibit neuroprotective actions, as KYNA can hardly penetrate BBB [10]. Furthermore, KYNA has been revealed to aid in leukocyte recruitment and regulation of anti-inflammatory actions in the neuronal tissue [96]. Numerous clinical and pre-clinical investigations have portrayed the significance of KYNA and its neuroprotective analogues in PD, by antagonizing the actions of NMDAR and retarding the excitotoxic actions in the neurons [10]. INF-γ production was found to be elevated by SN microglia in macaques administered with MPTP. Therefore, INF-γ is a significant KP inducer as well (Figure 2) [97]. Two more isotopes of KAT are reported, namely KAT3 and KAT4, where KAT3 is similar to KAT1 in genome structure, has high sequence homology, and exhibits significant specificity towards alpha-keto acids and L-amino acids, whereas KAT4 is similar to mitochondrial aspartate aminotransferase [98]. These NMDA antagonists can be significant psychopharmacological targets, due to which they grab the attention of the neuro-chemists [98].

**Figure 3:**
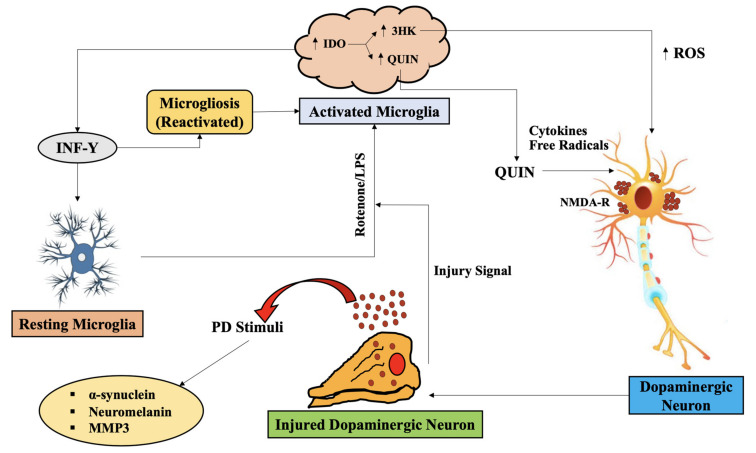
Microglial activation resulting in QUIN-mediated damage to the dopaminergic neuron, leading to PD. Legend: IDO—Indoleamine 2,3-dioxygenase, QUIN—quinolinic acid; NMDAR—NMDA receptor; KYNA—kynurenic acid, INF-γ—interferon-gamma, 3-HK—3-hydroxykynurenine, ROS—reactive oxygen species, PD—Parkinson’s disease, MMP3—matrix metalloproteinase-3., **↑** increased expression.

Additionally, QUIN was found to be produced and aggregated in macaque brain administered with MPTP by activated microglia upon co-localization with SN dopaminergic neurons [10]. Additional evidence and data support the microglia (activated) and NMDAR dopaminergic neurons in the SNpc region, which shows that NMDAR that is activated by elevated microglia-released endogenous QUIN as a result of the disease, and followed by neurotransmitter glutamate (excitotoxic), may contribute significantly in regulating the loss of dopaminergic neurons in PD (Figure 3) [99].

Numerous in vivo as well as in vitro investigations have further aided it, depicting the neuroprotective actions of antagonists of NMDA receptor against toxicity indued by MPTP. On the other hand, it was found that no NMDA receptor regulated neuroprotective effects after MPTP was injected via intra-striatal route [100]. Thus, the initial positive outcome occurs as a result of blockage of the system of reuptake of DA, when elevated MK-801 doses, antagonist of NMDA ion channel, are administered [10]. KYN is converted to its downstream metabolite, 3-HK (neurotoxic), under the action of kynurenine-3-monooxygenase (KMO), which, if pharmacologically inhibited, has been reported to promote KYNA production [101,102,103,104].

The dopaminergic metabolism in bipolar disorder, depression, and schizophrenia, has been reported to be impaired by KMO gene polymorphism [105]. The significant involvement of SNPs of KMO were investigated in PD [16,106]. However, the study was unable to recognize any relationship of the four SNPs investigated in PD and were unable to carry the binding sites for regulatory proteins, associated with PD pathogenesis [106].

Lim et al. elaborated on the involvement of KP in PD pathogenesis where the levels of KYN metabolites altered the levels of KP metabolites in L-DOPA treated PD patients, which influenced the glutaminergic transmission resulting in L-DOPA-induced dyskinesia (LID) [15]. The role of KP metabolites in LID was significantly studied, where the level of these metabolites was assessed in the CSF and plasma of PD patients with LID, and depicted an elevation of 4-folds in the ratio of 3-HK to KYNA levels and curbed AA levels [91]. QUIN infusions were used in rat brain, which retarded KYNA and KYN levels [107], further resulting in reduction in the limit of excitotoxicity. L-DOPA and D-amphetamine administration was reported to retard the levels of KYNA in rat brain [93,108].

The red blood cells (RBCs) and plasma of PD patients were evaluated for KYNA and the biosynthetic enzymes (KAT1 and KAT2), where enhanced KYNA levels along with KAT2 were observed, which may be a sequential pathway against the excitatory neurotoxic effects [109]. Furthermore, KYNA and KAT1 activity has also been reported to be responsible for 6-OHDA and MPTP toxicity. Similarly, the activities of biosynthetic enzymes were curbed in the plasma of patients with PD, with a reduction in KYNA, while increased levels of KYNA were related to elevated KATs in the RBCs of patients with PD [110]. Additionally, about 2–3-folds of DA levels in the striatum, is elevated by KAT-2 blockage, which can be protected by KYNA co-administration [95]. Further, an investigation on PD patients reported retarded TRP levels, with elevated ratio of KYN to TRP, increased KYNA, arachidonic acid (AA), and KYN, as compared to controls [111].

**Figure 4:**
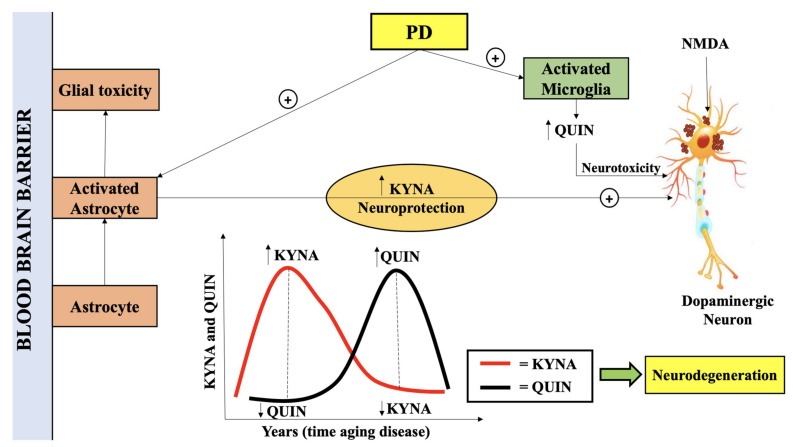
Neuroprotection mediated by KYNA against neurotoxic effects of QUIN, mediated by microglia and astrocyte activation. Legend: PD—Parkinson’s disease, NMDA—N-methyl-D-aspartate receptor, KYNA—kynurenic acid, QUIN—quinolinic acid, **↑** increased expression.

Further investigations with ameliorated KYNA levels were reported in caudate, cortical areas, SNpc, cerebellum, and putamen of PD patients [112]. Figure 4 depicts the antagonizing actions of KP metabolites QUIN and KYNA as a result of microglia and astrocyte activation.

The chemical reactions which constitute electron transfer between two molecular entities, are referred to as redox reactions. Redox metabolism comprises of a series of redox reactions, which are significant for removal of electrophilic oxidative species and harmful nucleophiles [113]. The imbalance generated between the production of reactive oxidizing metabolites and their excretion by the anti-oxidant or enzymatic processes results in the induction of oxidative stress, which is associated with numerous neurological disorders [113]. Multiple free radicals—such as hydroxyl radical and superoxide—as well as non-radical molecular entities—such as hydrogen peroxide—constitute the ROS species, which are produced by soluble intercellular components in the cytosol [114], where the enzyme XDH catalyzes xanthine to urate, NAD+ to reduced form, and NADH and water to hydrogen ion [113].

Nrf2 is a transcriptional factor of the anti-oxidative enzyme genes comprising of glutathione S-transferase, catalase, GPx, SOD, and GRx [113]. The Kelch-like ECH associated protein 1 (KEAP1) blocks the cytosolic UPS system, promoting translocation of Nrf2 into the nucleus to attach to the cis-acting enhancer sequence of the promoter region, the anti-oxidant response elements [115].

Another transcriptional factor of energy metabolism-regulating gene, peroxisome proliferator-activated receptor (PPARs), has been identified as reliable candidate for dis-orders such as diabetes [116]. Furthermore, numerous diseases are reported to be treated by polyphenolic antioxidants such as vitamin C, vitamin E, L-carnitine, folic acid, and N-acetyl cysteine. The neuronal disorders are reported to be ameliorated by polyphenols derived from olive leaves [117]. The Mediterranean diet is found to be effective against inflammation and curcumin is an antioxidant and anti-inflammatory agent, that prevents pain and stress via KP [113,114,115,116,117,118,119].

Certain endogenous oxidative and anti-oxidative KP metabolites are also significant in this regard [120,121,122,123], such as 3-HK (oxidative) and KYNA (anti-oxidative), which were found to be related to the depressive symptoms in stroke patients [118,124,125,126], where KYNA induced anti-depressant action in animal models of depression [127,128,129,130,131]. The onset age of neurodegeneration is regulated by IDO-1, which is related to depressive impact and inflammation [69,132,133,134].

Besides mediating excitotoxicity, QUIN elevates peroxidation of lipids in a Fe^2+^ and NMDAR-dependent manner [135,136,137,138]. Auto-oxidation is followed by the generation of highly reactive free radicals, as a result of iron complexes formation by QUIN. QUIN treatment at pathophysiological concentration leading to a dose-dependent elevation in the expression of inducible nitric oxide synthase (iNOS) as well as neuronal nitric oxide synthase (nNOS) in cultured astrocytes and neuronal cells in humans [139], which results in elevated toxicity and death of cells, determined by the absence of necessary pyridine nucleotide as well as the cofactor to produce adenosine triphosphate (ATP), NAD+. MK-801 mediated blockage of NMDA receptor or N-nitro-L-arginine methyl ester (L-NAME) mediated inhibition of activity of NOS, can retard cellular toxicity, depicting that production of nitric oxide (NO) is responsible for excitotoxicity, mediated by QUIN [140]. This is evidently supported by elevation in nNOS and iNOS, neuronal excitotoxicity, and lipid peroxidation in rat neuronal tissue, being the result of QUIN actions, which were curbed by NOS inhibition or antioxidants, such as U-83826E, melatonin, and α-phenyl-t-butyl nitrone [141]. Parkin modification mediated by NO, has been observed in post-mortem neuronal tissue in patients with PD, unlike the controls of similar ages and genders.

The production of non-functional and misfolded parkin is induced by oxidative stress, as detected in SNpc of patients with PD. S-nitrosylated parkin and nitrosative stress inactivate parkin functions, as reported by neuronal samples of sporadic PD patients [142]. This indicates the BBB-permeating effective NO scavengers can prove to be therapeutically useful in PD. QUIN mediated toxicity may also occur because of metabolic impairment. QUIN administration via intra-striatal route can facilitate reduction in ATP production and oxidative phosphorylation [30]. However, these actions might be because of glutamate receptor activation and production of free radicals. The reduction in actions of mitochondrial complexes 1 (50%), 2-3 (35%) and 3 (46%) of electron transport chain (ETC) in rat striatum is promoted by treatment with QUIN [30]. This action is considered to be partly mediated by ROS production. The familial and sporadic PD are characterized by mitochondrial dysfunction. Therefore, mitochondrial dysfunction is found to aid in enhanced vulnerability of DA neurons. These neuronal cells are subject to massive oxidative stress, on account of DA metabolism as well as excitotoxicity, whereas the antioxidant potential is narrow [143].

3-HK is another neurologically active KP metabolite, which can generate ROS. The cultured cortical as well as striatal neuronal cells when treated with 3-HK at pathophysiological levels have been reported to ameliorate outgrowths of neurites [144]. The antioxidant, catalase, can be used to prevent the morphological alterations but not superoxide dismutase (SOD). QUIN toxicity and development of neuronal lesions can be potentiated with co-injection of 3-HK via the intra-striatal route [145]. The antioxidants, such as N-tert-butyl-a-(2-sulpho-phenyl)-nitrone, and antagonists of NMDA receptor can abolish these abnormalities. The downstream KP metabolite, 3-HAA, promotes auto-oxidation, resulting in production of super-reactive oxygen species, which is substantially elevated by SOD, but curbed by catalase [119]. These results indicate that neurotoxicity, mediated by 3-hydroxy anthranilic acid (3-HAA) and 3-HK, occurs by production of free radicals, which can be prevented by pharmacological upregulation of catalase activity.

### 4.1. Upstream KP Metabolism in PD

KP metabolomics has provided a direct relationship between neurological conditions, specifically PD and psychoneuroimmunology [120,121], which aids in understanding the interlinking pattern between neuronal degeneration and inflammation of the neurons, where innate immunity contributes significantly to the progression of the disease and its pathophysiology. The chronic low-grade inflammation has been reported to promote KP activation [122] and is related to older people with changes in tyrosine metabolism and TRP levels. TRP is a significant precursor for serotonin–melatonin pathway, synthesis of proteins and KP metabolism, and creates a balance by regulating the pathways. However, during low-grade chronic inflammation, this balance is disturbed, throughout the activation of the immune system. As a result, the KP route catabolizes TRP, instead of the serotonin–melatonin pathway, resulting in the amelioration of the production of serotonin, leading to depression-like signs while the disease progresses [10]. A study extracted metabolites involved in the diagnosis of major depressive disorder and estimated the effect of escitalopram by using metabolomic approaches, where KYNA was identified among 73 metabolites for overlapped biomarkers [123]. The Hamilton Rating Scale for Depression (HRSD) was measured, where HRSD reduction was found to be negatively related to KYN and KYNA. Ameliorated levels of KYNA were found in major depressive disorder, which exhibited a better response to escitalopram [123]. The disease classification is enhanced and the exposure to treatments with limited efficacy is curbed by overlapping biomarkers that promote diagnosis of disease and prediction of therapeutic response [123]. Studies have identified a relationship between KP metabolites and depression, on account of immune response and activation of pro-inflammatory cytokines. The risk of depression was found to be elevated after immune-activating agents in critically ill patients, due to KP mediated actions [124].

Numerous published data provide suitable evidence, suggesting the involvement of QUIN in mood and behavioral disorders, via its actions on the NMDA receptors [125,126]. The translational validity in PD pathogenesis is reported by dysregulated immune system and disturbed neurological behavior. The levels of pro-inflammatory markers such as, MCP-1, IL-6, and C-reactive protein (CRP) are found to be elevated in the CSF of PD patients, which are related to the non-motor PD symptoms, such as cognitive disruption, tiredness and depressive behavior [127], which may promote the occurrence of non-motor signs. This process is associated with sickness behavior, that is stimulated by elevated KP activation. Thus, IDO-1 activation, mediated by INF-γ—which can be promoted by other cytokines, such as TLR agonists and IL-1β—may also take place in PD [10].

The elevated TRP catabolism, accordingly displayed by the ratio of KYN/TRP, exhibits positive relationship with neopterin (the inflammatory marker) in the CSF and periphery of patients with PD, which further provides a proof to the immune system mediators, promoting activation of KP in patients with PD [25]. These trends are associated with progression of the disease in PD cohort depicting those metabolic changes in KP that may enhance complexity of the disease in patients. Pycnogenol (PYC) was used as an anti-inflammatory compound to curb the activation of microglia and neuroinflammation, as well as to provide protection to the dopaminergic neurons against toxicity induced by MPTP and impaired mitochondrial functions [128]. The expression level of nuclear factor-kappa B (NF-κB) is hindered in PD mice model administered with MPTP as a result of PYC therapy, which implicates IDO-1 suppression in monocytic cells, especially microglia, as NF-κB being a regulator of upstream metabolic processes, inducing IDO-1 expression [129]. The activation of IDO-1 in microglial cells is related to degeneration of neurons and psychosis [130]. Thus, IDO-1 blockage serves as a target site for PD treatment, that shall be subject to future considerations.

### 4.2. Downstream KP Metabolism in PD

Multiple downstream neuroactive metabolites are produced as a result of KP activation in PD. KYN production results in downstream generation of QUIN instead of KYNA, particularly during immune challenge, comprising of microglial cells that are mostly present in association with PD. QUIN is a selective NMDA agonist, as discussed earlier, especially with NR2A and NR2B subunit containing NMDAR subtypes with massive entry of astrocytes and neuronal cells [131]. Thus, QUIN-mediated toxic effects are highest in the presence of these receptor types. The regions relevant to PD pathogenesis, such as gamma amino benzoic acid (GABA) and substance P, containing striatal spiny neurons exhibit vulnerability towards QUIN toxicity. The slight variation of QUIN levels above the physiological concentration can promote induction of neuronal degeneration in cortico–striatal cell culture in rats [132]. Significant behavioral alterations were induced, when QUIN was administered via intra-striatal route [133]. QUIN lesions were reported to be followed by cognitive deficiency in rats, which showed that QUIN can impair spatial reference memory [134]. Additionally, the performance of rats on balance beam, open field tasks and radial arm water maze, was found to be curbed, as the result of QUIN administration. Apomorphine (DA agonist) administration resulted in development of unilateral QUIN lesions and behavioral abnormalities in rodents, which occurred due to imbalance in dopaminergic signaling between the normal and damaged hemisphere [135]. Moreover, axon-sparring lesions were also reported to occur as a result of intra-striatal QUIN injections, which also promoted swelling of dendrites and absence of structural characteristics of cells at post-synaptic sites, as per the post mortem analysis reports; however, the axons close to the presynaptic terminals were found to be largely preserved [136].

KYNA, another significant KP metabolite, exhibits antioxidant characteristics, on account of its ability to act as a free radical scavenger for superoxide anions and hydroxyls. KYNA antagonizes the actions of a7-nicotinic acetylcholine (a7nACh) receptors, non-competitively, at normal physiological concentration, and regulates the level of glutamate and DA as well as acetylcholine in the CNS [71]. KYNA antagonizes the actions of α-amino-3-hydroxy-5-methyl-4-isoxazolepropionic acid (AMPA), kainic acid (KA), and NMDA receptors, at high micromolar concentrations, and curbs the neurotoxic effects mediated by QUIN. The shifting of KYN catabolism either towards QUIN or KYNA, must be significantly modulated under normal physiological conditions in order to promote efficacy of neuroprotective metabolites in actions of downstream neurotoxic TRP metabolites [10,137]. Antagonists of AMPA and NMDA receptors have been reported to alleviate the development of motor symptoms, induced by L-DOPA in rats [100].

Glutamate release and toxicity is inhibited by the antagonizing actions of KYNA on AMPA, KA, and NMDA receptors. The metabolic arm of KYN is shifted towards KYNA by the action of RO 61-8048, which is a KMO inhibitor, thereby elevating KYNA levels and ameliorating dyskinesia in MPTP monkeys with LID [102]. It is noteworthy, that KP activation does not always tend to induce damaging results, as if the pathway is shifted towards KYNA production, it might exert a positive impact, which was evidently supported by prevention of cell death, promoted by MPP+, by pretreatment with KYNA in dopaminergic neuronal cell lines in humans [138]. Furthermore, direct injection of KYNA into globus pallidus internus in PD patients provided protection against toxicity induced by MPTP [139,140].

Additionally, extraordinarily little therapeutic efficacy is observed because of systemic administration of exogenous KYNA on account of its limited penetration ability across BBB as well as short-lived half-life. Furthermore, antagonistic action at the glycine region in non-human primates as well as rodent LID-PD models, has been predicted to provide protection against the excitotoxic consequences in PD [141]. However, blockage of ionotropic glutamate receptors can further propagate harsh consequences, such as cognitive dysregulation in multiple patients.

### 4.3. Microbiota Gut Brain Association with KP in PD

Despite the localization of neurological disorders in the brain, the researchers have depicted a significant relationship between the gut and the brain. The accumulation of α-synuclein in PD begins in the gut, followed by dissemination to the neuronal tissue, via the vagus nerve [142]. The Lewy bodies are the inclusions of α-synuclein protein which have been observed in maximum amounts in submandibular gland and lower part of the esophagus, and also contained in stomach, rectum, small intestine, and colon [143]. As the age grows, the chances of development of α-synuclein also increases [144]. Accumulation of α-synuclein and neurodegeneration is associated with constipation, which is a non-motor PD symptom that is accompanied by elevated signs of intestinal permeability, oxidative stress, and inflammation [145,146]. Such pathological alterations precede the motor symptoms associated with PD, which provides a significant evidence of initiation of PD pathogenesis in the gut.

The gut microbiota is considered to exert a stringent effect on the KP metabolites, as per the results obtained from multiple investigations. The KYN in the gut has the ability to penetrate the BBB and aid in the development of KP metabolites in the neuronal tissue [147]. In the neuro gastroenterology investigations, KYNA and QUIN are regarded as the most significant KP metabolites. Even though, their exact functions are not fully known, yet they function as immunoregulator agents [148]. Immunoregulatory actions and gastric mucosal defense mechanism is regulated by KYNA in the gastrointestinal tract, via G-protein coupled receptor (GPCR), i.e., GPR35 [149]. The KP associated enzyme are encoded by multiple intestinal bacteria, which result in the production of 3-HAA and KYN [150], which exerts neurotoxic effects during diseased states [151]. Microbial colonization [152] has been observed to be the cause of changes in the plasma levels of KP metabolites under diseased states [151,153,154,155]. Certain investigations have suggested generation of metabolite assemblies, promoting accumulation of α-synclein, as a result of excess QUIN due to mutations in the KP [156]. Previous investigations have portrayed the association between gut microbiota and KP in neurodegenerative diseases [157,158]. Such a link has attracted significant attention, as it may serve as a suitable therapeutic target for PD treatment in the future.

## 5. Applicability of KP as A Biomarker in PD

Understanding the factors, which lead to the development of PD and other neuropsychiatric disorders would aid in promoting healthy aging and prolonged health in geriatric patients. Therefore, validation and accessibility of biomarkers from pathological and normal subjects is a chief requirement in this regard. Metabolomics is a significant approach which complements hypothesis driven techniques and targets. Specific moieties, with known associations, such as molecular signals, such as α-synuclein, are considered to be a significant target in PD patients [10]. However, numerous biochemical pathways, lipid and cholesterol metabolism, inflammatory processes, proteolysis, dysfunction of BBB, metal ion homeostasis, and structure of the cytoskeleton, regulate a multifactorial disorder such as PD. Yet, most of these processes occur as a result of normal brain aging, and there is a requirement to provoke the aging process, to be a programmed process, determined by genetics, and disorders associated with distinct entities with individual risk factors [10].

CSF examination is a conventional technique, used to investigate disorders related to the brain, which are responsible for bathing the brain and carry out its protein signaling processes. As discussed previously, regarding the role of changes in the metabolic pathway of KP in PD, these metabolites are reported to indicate biochemical processes specific to the disease [10]. CSF samples were collected before 4 h postmortem, from 48 pathological patients of PD and 57 controls of comparable ages and were assayed by employing ultra-high-performance liquid as well as gas chromatography, in association with mass spectrometry [159]. A total of 19 biochemicals out of the 243 identified compounds differentiated control subjects from patients with PD at a false discovery rate of 20% [159]. The concentration of 3-HK was found to be elevated by one-third level as well as mean oxidized glutathione was found to be ameliorated by 40% in PD patients [159]. Such conclusions evidentially confirm the role of excitotoxicity and KP in PD progression. Investigation of optimistic disease-specific association between metabolomic evaluation of blood samples and CSF biomarkers is a matter of future considerations.

Multiple biological fluids are reported to be suitable candidates for biomarker detection, such as CSF, urine, and blood [160]. The exact cellular alterations in affected neuronal tissue of PD patients was reflected by biofluids (serum and CSF), which were collected alongside complete assessment of phenotypic features from PD patients [161]. It was reported that KP related compounds were able to cross the BBB easily and enter the CSF [9,162]. The KP metabolites are reported to be related to PD progression, either directly or indirectly, along with variations in the blood.

Elevated IDO levels were reported in the blood of PD patients, resulted during the aging process [163]. In fruit flies, with expressed α-synuclein, the ratio of KYN to KYNA, and urine samples of PD patients were found to be elevated [164]. The urine samples of PD patients were reported to exhibit 18 different metabolites, in which the concentration of KYN was found to be increased [165]. Enhanced 3-HK and KYN concentrations were reported in the CSF in another investigation, which may aid in the induction of oxidative stress in patients with PD [166]. The concentration levels of KYN to TRP, along with AA, KYNA, and KYN levels, were found to be enhanced in PD patients [8].

QUIN to KYNA ratio and concentration of QUIN was reported to be elevated in the plasma samples of PD patients [167]. KYNA levels were reported to curbed in the CSF samples, with elevation in KYA, QUIN and KYN levels in the serum, as per KYA pathway study in PD [8]. All these findings depict the significance of biomarker studies, in order to recognize the initial signs and symptoms associated with PD and further profiling which is useful for the development of suitable pharmacological intervention. Furthermore, urine KYN was found to be related to severity in PD patients, along with mild cognitive impairment. Therefore, urine KYN may function as a novel biomarker for early stage diagnosis of PD. Table 1 lists the biomarker studies in biological fluids, exerting notable actions on KP metabolites in PD.

## 6. Exploring the Therapeutic Role of KP in PD

Numerous therapeutic approaches have been investigated to elevate the levels of endogenous KYNA or to alleviate the production of QUIN. In AD animal models, it was used as pharmacological treatment, to retard the PD progression and related disorders. Co-administration of L-KYN is the prime precursor for KYNA, which when co-administered with organic anion transporter inhibitor, was reported to elevate KYNA, resulting in reversal of excitotoxicity induced by glutamate in rats with PD, induced by 6-OHDA [110]. Besides regulating the release of glutamate from cortex to the striatum, elevation in KYNA resulted in direct antagonizing action on NMDA receptors, thus reducing glutamate excitotoxicity. KYNA and L-KYN analogues were developed to overcome the limited half-life of these main metabolites [10]. These analogues included 7-chlorokynurenic acid (7-Cl-KYNA), L-4-chlorokynurenine (4-Cl-L-KYN), and 2-(2-N,N dimethyl amino ethyl amine-1-carboyl)-1H-quinoline-4-one hydrochloride [168,169,170].

These analogues have been developed to elevate the pharmacological characteristics and stability profile of the main metabolites. 4-Cl-L-KYN has the potential to penetrate BBB and hinder toxicity mediated by QUIN at the glycine site on the NMDA receptor [171]. Considering BBB permeability, the analogues of KYNA also exhibit the ability of inducing glutamate suppression as well as NMDA activation, thereby gaining therapeutic importance in PD, which is yet to be evaluated. D-glucose or D-galactose, in combination with KYNA analogues, result in increased ability to cross BBB and prevent and curb the chances of seizures and excitotoxicity in animal models [172].

Improvement in the motor symptoms in PD patients with LID, was observed on treatment with zonisamide, which is a sulfonamide anti-epileptic drug, although, the mechanism is not yet known. However, zonisamide has been reported to elevate KYNA production [10], which further provides an evidential data related to the involvement of KYNA in treating PD-associated LID. It has been reported that chronic and acute exposure of astrocytes to zonisamide results in KYNA generation as well as other neurologically active KP metabolites, such as CA and XA [173], where both of these metabolites have been identified as endogenously produced agonists of first and third group of metabotropic glutamate receptors [174,175].

Group 2-mGluR and Group 3-mGluR activation are reported to function as significant targets of drug candidates to provide symptomatic relief as well as neuroprotection in patients with PD, besides KYNA, as per numerous preclinical investigations. Thus, these two prime metabolites of KP may possess therapeutic significance in PD treatment [176,177]. An INF-mediated elevation in CA was reported that result because of chronic exposure to zonisamide, which also induced reduction in the production of QUIN [173]. Therefore, this results in shifting of the ideal paradigm towards elevated neuroprotection provided by XA, KYNA, and CA, while the excitotoxicity exhibited by QUIN is yet at bay.

A KP associated enzyme, KMO, which, when blocked can result in ameliorating of generation of downstream metabolites by 3-HK, can further induce diversion of KYN towards generation of KYNA [10]. The progression of dystonia in hamsters is significantly retarded by KMO inhibitors thus exhibiting its role as potential agents for treating dyskinesia, related to dysfunction of striatum [178]. The KMO inhibitors, such as Ro61-8048, have been reported to decrease dyskinesia induced by L-DOPA in monkeys administered with MPTP, with no comprise in the therapeutic role of L-DOPA, as well as elevated production of KYNA [102,179]. Furthermore, KMO inhibitor nicotinyl alanine, in combination with probenecid and KYN, was observed to increase the production of KYNA, against excitotoxicity induced by QUIN and NMDA in nigrostriatal dopaminergic neurons [107]. Such a protective action does not exhibit any specificity towards KYNA enhancement, as administration of precursor alone, with no pharmacological intervention does not provide any beneficial actions in PD treatment in animal studies [10].

A neuro immunophilin ligand, FK506, is employed as an immune system suppressing agent in PD treatment, which besides elevating KYNA production in the cortex, also reduces KYNA inhibition, which is carried out by 3-nitropropionic acid and MPP+ [180]. Certain investigations have depicted that treatment with this ligand could elevate the survival chances of DA neurons in a dose dependent manner but therapy for a short period of time only exerts a minor impact on the progression of the disease [181]. Furthermore, infiltration of cytotoxic T cells and T-helper cells, along with subtypes and number of macrophages and microglial cells were significantly retarded as the result of FK506 administration [181]. Such data depicts that FK506 exhibits anti-inflammatory functions, which resulted in reduction of neurodegeneration and portrays its role in PD treatment.

## 7. Identifying other therapeutic targets of PD

Numerous targets have been identified to facilitate PD treatment, such as chaperones, protein Abelson (c-Abl), glucocerebrosidase-1 (GBA-1), calcium, neuromelanin, ubiquitin-proteasome system (UPS), neuroinflammation, mitochondrial dysfunction, and KP. Chaperones are the protein entities that are elevated as a response to temperature, due to which they are also referred to as heat shock proteins (HSP) [2,106]. These regulate folding, refolding and degradation of proteins, thereby sustaining proteostasis. Overexpression of HSP70 was reported to retard α-synuclein mediated neurodegeneration of dopaminergic neurons [182,183]. The development of PD is aggravated in states where HSP70 in mitochondria exhibits mutation [2]. Furthermore, HSP70 has been reported to prevent death of dopaminergic neurons, promoted by 1-methyl-4-phenyl-1,2,3,6-tetrahydropyridine (MPTP) toxin, in PD models [184].

Currently, multiple drug candidates are undergoing clinical trials targeting chaperone and show promising results in PD. An orally administered, mucolytic therapeutic compound, ambroxol hydrochloride, functions as a chaperone of pharmacological significance for mutant glucocerebrosidase and certain studies have shown its action in retarding PD [2]. The c-Abl is a member of tyrosine kinase family, reported to be stimulated by cellular as well as oxidative stress. Inhibitors of c-Abl have the ability to cross the BBB and provide protective action against toxicity, induced by MPTP in mouse model of PD, by reversing the loss of dopaminergic neurons with noticeable improvement in motor symptoms [185]. It is known that a c-Abl inhibitor, nilotinib, which is used to treat chronic condition of leukemia is under investigation for PD treatment. Another target for PD treatment is GBA-1 gene, encoded on glucosidase enzyme, which is produced in rough endoplasmic reticulum.

Oxidative stress is often considered to be one of the most significant pathological hallmarks of PD, as confirmed by post-mortem evaluation of patients with PD. The reactive oxygen species (ROS) are consistently produced by all cells via respiratory chain, and function as secondary messengers in cell signaling, alongside normal physiological roles at limited or medium levels [186]. However, exposure for a longer period of time or severe ROS levels can disrupt the prime macromolecules of the cells, such as proteins, lipids, and deoxy-ribonucleic acid (DNA), resulting in cell death via necrosis and apoptosis [187]. The disrupted balance between ROS production and impaired natural antioxidant system creates the condition of oxidative stress in the body, which among all the body systems severely damages the CNS due to its elevated oxygen demand, terminal differentiation of neuronal cells, and weak antioxidant system, because of which oxidative stress forms the basis of numerous neurodegenerative disorders in the body [186].

Extensive research has been carried out to investigate potential antioxidants to ameliorate oxidative stress conditions, such as free radical scavenging vitamin D (water soluble antioxidant); vitamin C, exhibiting neuroprotective actions against L-DOPA induced neurotoxicity [186]; mangosteen pericarp water extract exhibiting antioxidative neuronal protection and anti-apoptotic effects in mice, resulting in enhanced spatial memory [188]; MAO-A inhibitor, Verbascoside [189]; essential oil of *Thymus vulgaris* [190,191]; and *Juglans regia* L. extract, which mitigates oxidative stress in neuronal tissue and enhances cognitive potential [186].

Enhanced ROS levels were found in fibroblasts of PD patients, which compromised GBA1 mutation as compared to the wild type GBA1 [2]. Currently, numerous clinical trials are taking place depicting the role of GBA as a biomarker in PD. Furthermore, neuromelanin is brain melanin, which comprises of eumelanin surface and pheomelanin core. The production of neuromelanin constitutes the antioxidant pathway, resulting in transforming oxidized DA products (toxic), such as quinone and semiquinone, into neuromelanin [2].

Significant information has been collected for the relevance of in vivo administered iron and neuromelanin as quantification tools for diagnosis of dystonia associated with PD, but the data related to it has not yet been fully investigated. Synaptic vesicles (SV) are contained in the synaptic buttons, which act as storage units of neurotransmitters, where the SV2 family comprise of 12 transmembrane proteins with 3 major families, SV2A, SV2B, and SV2C [2]. The SV2C family has been reported to be present in basal ganglia and has been observed to be used in multiple neurological disorders, such as PD, AD, and epilepsy. Studies indicated alterations in the SV2C expression in mouse models, which resulted in loss of dopaminergic cells. Some investigations reported the regulation of protective actions of nicotine in PD by alterations in the SV2C gene variation [192]. Further, calcium can be found in all the organisms and functions as a secondary messenger, where PD pathophysiology is reported to be significantly associated with L-type calcium channels.

In juvenile SNpc dopaminergic neurons, calcium channel, voltage dependent, L-type, α1C-subunit (Cav1.2) is present in active form, whereas during aging it is used by dopaminergic neurons to promote calcium influx, thereby aiding rhythmic pacemaker activity, which is significant in the maintenance of the striatal dopaminergic levels as highlighted by the role of Cav1.2 in regulation of dopaminergic firing activity in the ventral tegmental area in PD mouse models [193,194,195]. Furthermore, MPTP intoxicated Cav1.2 knockdown mice exhibit degeneration of dopaminergic neurons, which elaborates the detrimental effects of inhibition of Cav1.2 on PD [196]. Cav1.3 is reported to be an effective therapeutic target for PD. Cav1.3 L type calcium channel (LTCC) promotes calcium influx, during autonomous pace making in SNpc dopaminergic neurons in adults, which is associated with mitochondrial oxidative stress in preclinical models [197].

LTCCs play a significant role in regulating firing activities in the ventral tegmental area in mouse PD models. Distinctive functions of Cav1.2 and Cav1.3 have been elaborated, during regulation, in two transgenic strains of mice [198]. The mice defiant in Cav1.3, exhibited a lower frequency of basal firing. Administration of nifedipine (dihydropyridine channel blocker) ameliorated single spike firing in dihydropyridine-insensitive Cav1.2 channel mice, which confirms the essential role of Cav1.3 subtype in basal firing [198]. Further, the firing patterns were converted from single spiking to bursting in mice expressing dihydropyridine-insensitive Cav1.2 channels, which was hindered by nifedipine depicting the significant role of Cav1.3 in burst firing also [198]. Therefore, these findings lead to important conclusions regarding the significance of Cav1.3 in regulating basal single-spike firing, whereas both Cav1.2 and Cav1.3 activation aids burst firing of neurons in the ventral tegmental area [198]. Investigations have revealed the significant involvement of oxidative stress (OS) conditions in PD progression; however, the mainstream process is not yet clear. Despite being the source for reactive oxygen species (ROS), mitochondria also serve as a target for ROS, which portrays the role of mitochondrial dysfunction in PD pathogenesis [113,199]. Mitochondria plays a significant role in production of energy, cellular homeostasis, cellular death, and stress response, due to which any impairment or damage to the organ results in neuronal degeneration. ROS is primarily produced from the superoxide anion generated in the ETC, where the electron transport processes aid in the synthesis of ATP, via ATP synthase, i.e., complex 5 [2]. The superoxide anion is also generated by other complexes, namely complex 1, 2, and 3, during the functioning of the ETC. NAD+ (complex 1) is a ubiquinone oxidoreductase enzyme, which produces ubiquinol from ubiquinone by energy extraction from oxidation of NADH, where ubiquinol functions as a carrier within the membrane for the pair of electrons followed by their transfer to complex 3 [2]. The electrons are discharged through uniquinol to complex 3 by complex 2, i.e., succinate-coenzyme Q reductase, which aids to establish a relationship between ETC and Krebs cycle [2]. Cytochrome C is reduced by ubiquinone cytochrome C oxidase (complex 3), via ubisemiquinone oxidation, followed by pumping of proton from mitochondrial matrix into the inner membrane space of the organelle. Reduction in the electron transfer in the ETC results in capture of electron by molecular oxygen from ubiquinone cytochrome C oxidase, resulting in the formation of superoxide anion [2].

In the MPTP-induced PD, loss of dopaminergic neurons was revealed by post-mortem analysis, in which after penetrating the BBB, MPTP is metabolized into 1-methyk-4-phenylpyridinium (MPP+), by monoamine oxidase B (MAO-B), followed by the use of MPP+ by dopaminergic neuronal cells, resulting in blockage of complex 1 of the ETC [2]. Furthermore, a reduction in complex 1 and ubiquinone in PD patients has been reported; this leads to degeneration of the neurons. Mitochondrial dysfunction in PD patients was confirmed by gene profiling of dopaminergic neurons and also differentiates between mitochondrial fusion/fission cycle and mitophagy [2,200]. Loss of ATP13A2, elevated mitochondrial mass to enhanced oxygen consumption are reported in certain patients, associated with PD, which results in increased production of ROS in cell culture [201].

**Figure 5:**
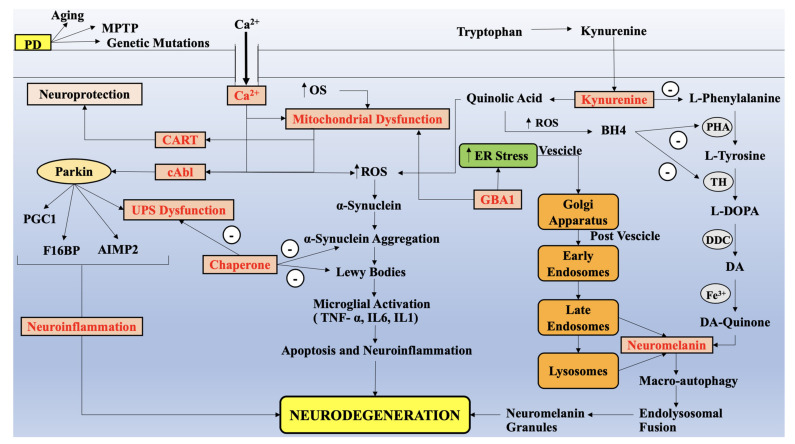
A mechanistic approach of potential targets for the treatment of Parkinson’s disorder (PD). The production of reactive oxygen species (ROS) is initiated by genetic mutations, aging, chemicals, etc., via mitochondrial dysfunction, which intervenes with ubiquitin proteasome system (UPS) and α-synuclein, resulting in the production of Lewy bodies. These further interfere with activation of microglial cells resulting in neuroinflammation and cell death. Calcium influx stimulates mitochondrial dysfunction, which further stimulates protein Abelson (c-Abl), further stimulating Parkin, followed by UPS dysfunction, PGC1, F16BP, and AIMP2. Mitochondrial dysfunction is also stimulated by glucocerebrosidase-1 (GBA-1), which elevated ER stress and aids in formation of neuromelanin pigments, resulting in neurodegeneration and cell death. Kynurenine is transformed to quinolic acid, stimulating tetrahydrobiopterin (BH4) and ROS, where the former blocks dopamine (DA) synthesis by hindering the enzymes, tyrosine hydroxylase (TH), and phenylalanine hydroxylase (PHA). Cocaine–amphetamine regulated transcript (CART) provides neuroprotection by protecting DA neuron. Chaperone binds to α-synuclein and blocks activation of microglia, production of Lewy bodies and UPS dysfunction, ameliorating production of proinflammatory cytokines, preventing cell death. Legend: PD—Parkinson’s disease, ROS—reactive oxygen species, UPS—ubiquitin proteasome system, c-Abl—protein Abelson, GBA-1—glucocerebrosidase, BH4—tetrahydrobiopterin, TH—tyrosine hydroxylase, PHA—phenylalanine hydroxylase, CART—cocaine–amphetamine regulated transcript, DA—dopamine, MPTP—1-methyl-4-phenyl-1,2,3,6-tetrahydropyridine, OS—oxidative stress, Ca^2+^—calcium ions, PGC-1—peroxisome proliferator-activated receptor-gamma coactivator 1, AIMP2—aminoacyl t-RNA synthetase-interacting multi-functional protein 2, TNF-α—tumor necrosis factor, IL—interleukins, ER—endoplasmic reticulum, Fe^3+^—ferric ions, DDC—dopa decarboxylase, **↑** increased expression.

Alteration in the level of miRNAs has been found to be related to ROS formation as well as impairment of mitochondrial function, which in turn plays a significant role in progression of neurodegenerative disorders [202]. Metabolic dysfunction and glial activation in the hypothalamus are found to result in negative energy balance, which significantly aids in neurodegeneration [203]. At present, various clinical trials are investigating functional and metabolomic biomarkers for mitochondrial dysfunction in PD. The ubiquitin–proteasome contributes significantly to the degradation of cells, followed by their removal, alongside elimination of unsolicited proteins [2]. Significant components associated with UPS include genes such as, ubiquitin carboxy-terminal esterase L1 and parkin, where mutations in these genes facilitate the development of a significant link between UPS and PD pathogenesis, as well as the role of UPS in the regulation of degradation of TH, which widens the understanding of PD pathogenesis [204,205]. Even though, multiple pre-clinical studies have been carried out to evaluate the impact of UPS in pathological events of PD, none of them has yet been carried out on human subjects. Figure 5 portrays different therapeutic targets for PD treatment.

Various investigations evidently support the involvement of neuroinflammation in PD progression, which is primarily adjudicated by microglia activation, being a source of superoxide it aids in the development of oxidative stress conditions in the neuronal tissue [206,207] and produces tumor necrosis factor-alpha (TNF-α) and glutamate, which in turn supports neurodegeneration. Microglia promotes neuroprotection by removing endogenous and exogenous substances and has elevated levels of glutathione, that provide protection from excessive levels of hydrogen peroxide [2]. Therefore, microglia serves a dual purpose in the neuronal tissue. Carrageenan-induced peripheral inflammation, followed by its administration in rat paw, deteriorates inflammation, induced by lipopolysaccharides, alongside the loss of dopaminergic neurons [2]. A N-methyl-D-aspartate (NMDA) receptor antagonist, memantine, has been reported to exert a protective action P2 × 7 purinergic microglial receptor, promoting activation of NLR Family Pyrin Domain Containing 3 (NLPR3) α-synuclein in murine model, which further triggers neuroinflammation contributing to deterioration of neuronal cells [2].

## 8. Conclusions

A vast amount of population is currently affected by neurodegenerative disorders, which is the most prevalent in the geriatric patients, among which PD and AD are the most common ones. Despite the conventional therapeutic paradigm employed in PD, the results exhibit limited efficacy, lesser tolerance, elevated side effects, and progressive deterioration of the dopaminergic neurons, where the ratio of positive to negative effects keeps on decreasing. As a result, a more reliable approach is on demand, with more optimistic drug targets. The manuscript provides a detailed account of KP, which is a TRPmetabolism pathway and its role on PD, with the help of the downstream and upstream metabolic processes, which collectively modulate the events related to the disease. The two major metabolites involve QUIN (neurotoxic), which acts via NMDA receptor, and KYNA (neuroprotective), which exhibit antagonizing actions to each other, therefore advocating the Janus-faced role of KP metabolites in PD.

The authors also display the role of KP as a biomarker, and involvement of other KP metabolites and enzymes such as 3-HK, 3-HAA, TRP, etc. in PD pathogenesis. Based upon the dual actions, the therapeutic possibilities in the disease are inferred, which has led to the development of multiple drug candidates targeting KP metabolites in PD. The authors aim to bring into light the possibility of targeting KP as a therapeutic candidate, to facilitate development of promising treatment approaches, providing an opportunity for the researchers to carry out further investigations in this regard to establish possible development of PD treatment in the future of neurological healthcare.

## 9. Future Prospects

The complexity of neurodegenerative disorders makes it difficult and also necessary to evaluate the exact mechanism behind the neurological disorder. Due to elevated risks and mortalities associated with brain disorders, the clinical researchers in this context are carried out on animals and not on humans. However, there is a need for clinical studies on humans, to study more the effects of therapeutic targets to facilitate disease mitigation. The researchers should emphasize of development of potential drug candidates targeting suitable sites, metabolites, or enzymes of KP. Despite the methods of either blocking the QUIN roads or opening the KYNA pathway, the researchers should investigate greater number of routs in KP leading to PD treatment. Another challenge is exploring more reliable ways to deplete side effects and exhibit greater effectiveness. An inhibitor, capable of crossing the BBB, is greatly desirable on account of its capability to prevent aggregation of neurotoxic metabolites and providing neuroprotection by elevating KYNA levels in the brain [53]. Additionally, most of the KMO inhibitors function as effectors, instead of a true inhibitor, due to which flavin reduction is promoted along with production of hydrogen peroxide, which is cytotoxic [53]. Therefore, such inhibitors should be avoided in the future. Thus, varying disease study models are required for validation of therapeutic tendency of candidates in the future along with inhibitor modification to get more effective compounds in PD treatment.

## Figures and Tables

**Table 1:** Representation of studies of biomarkers in bio-fluids and their effects on KP metabolites in PD.

Biofluid Biomarker	Metabolites	Observation	Ref.
Serum	Kynurenines	Decreased concentration of TRP	[8]
Urine	Urinary metabolites	Elevated α-synuclein and modified metabolism of tryptophan	[8]
CSF and serum	No metabolites mentioned	Decreased KYNA in CSF; increased KYNA, KYN and QUIN in serum	[8]
Plasma	184 metabolites	Elevated KYN/TRP ratio, KYN, AA, KYN	[108]
		Increased QUIN/KYNA ratio	[167]
		Increased QUIN	[8]
CSF	No metabolites mentioned	Increased KYN and 3-HK	[166]

## Data Availability

Not applicable.

## References

[1] Berg D., Postuma R.B., Bloem B., Chan P., Dubois B., Gasser T., Goetz C.G., Halliday G.M., Hardy J., Lang A.E. (2014). Time to redefine PD? Introductory statement of the MDS Task Force on the definition of Parkinson’s disease. Mov. Disord..

[2] Pingale T., Gupta G.L. (2021). Current and emerging therapeutic targets for Parkinson’s disease. Metab. Brain Dis..

[3] Tepper S., Ashina M., Reuter U., Brandes J.L., Doležil D., Silberstein S., Winner P., Leonardi D., Mikol D., Lenz R. (2017). Safety and efficacy of erenumab for preventive treatment of chronic migraine: A randomised, double-blind, placebo-controlled phase 2 trial. Lancet Neurol..

[4] Angot E., Brundin P. (2009). Dissecting the potential molecular mechanisms underlying α-synuclein cell-to-cell transfer in Parkinson’s disease. Parkinsonism Relat. Disord..

[5] Bartel W.P., Van Laar V.S., Burton E.A., Gerlai R.T. (2020). Chapter 23—Parkinson’s disease. Behavioral and Neural Genetics of Zebrafish.

[6] George J., Mok S., Moses D., Wilkins S., Bush A.I., Cherny R.A., Finkelstein D.I. (2009). Targeting the progression of Parkinson’s disease. Curr. Neuropharmacol..

[7] Jankovic J., Tan E.K. (2020). Parkinson’s disease: Etiopathogenesis and treatment. J. Neurol. Neurosurg. Psychiatry.

[8] Venkatesan D., Iyer M., Narayanasamy A., Siva K., Vellingiri B. (2020). Kynurenine pathway in Parkinson’s disease—An update. Eneurologicalsci.

[9] Schwarcz R., Bruno J.P., Muchowski P.J., Wu H.-Q. (2012). Kynurenines in the mammalian brain: When physiology meets pathology. Nat. Rev. Neurosci..

[10] Lim C.K., Fernandez-Gomez F.J., Braidy N., Estrada C., Costa C., Costa S., Bessede A., Fernandez-Villalba E., Zinger A., Herrero M.T. (2017). Involvement of the kynurenine pathway in the pathogenesis of Parkinson’s disease. Prog. Neurobiol..

[11] Munn D.H., Mellor A.L. (2013). Indoleamine 2, 3 dioxygenase and metabolic control of immune responses. Trends Immunol..

[12] Opitz C.A., Heiland I. (2015). Dynamics of NAD-metabolism: Everything but constant. Biochem. Soc. Trans..

[13] Castellano-Gonzalez G., Jacobs K.R., Don E., Cole N.J., Adams S., Lim C.K., Lovejoy D.B., Guillemin G.J. (2019). Kynurenine 3-monooxygenase activity in human primary neurons and effect on cellular bioenergetics identifies new neurotoxic mechanisms. Neurotox. Res..

[14] Erhardt S., Schwieler L., Imbeault S., Engberg G. (2017). The kynurenine pathway in schizophrenia and bipolar disorder. Neuropharmacology.

[15] Stetler R.A., Leak R.K., Gan Y., Li P., Zhang F., Hu X., Jing Z., Chen J., Zigmond M.J., Gao Y. (2014). Preconditioning provides neuroprotection in models of CNS disease: Paradigms and clinical significance. Prog. Neurobiol..

[16] Török N., Török R., Szolnoki Z., Somogyvári F., Klivényi P., Vécsei L. (2015). The genetic link between Parkinson’s disease and the kynurenine pathway is still missing. Parkinsons Dis..

[17] Houser M., Tansey M. (2017). The gut-brain axis: Is intestinal inflammation a silent driver of Parkinson’s disease pathogenesis?. NPJ Parkinsons Dis..

[18] Massudi H., Grant R., Guillemin G.J., Braidy N. (2012). NAD^+^ metabolism and oxidative stress: The golden nucleotide on a crown of thorns. Redox Rep..

[19] Castro-Portuguez R., Sutphin G.L. (2020). Kynurenine pathway, NAD^+^ synthesis, and mitochondrial function: Targeting tryptophan metabolism to promote longevity and healthspan. Exp. Gerontol..

[20] Cervenka I., Agudelo L.Z., Ruas J.L. (2017). Kynurenines: Tryptophan’s metabolites in exercise, inflammation, and mental health. Science.

[21] Liu M., Wang X., Wang L., Ma X., Gong Z., Zhang S., Li Y. (2018). Targeting the IDO1 pathway in cancer: From bench to bedside. J. Hematol. Oncol..

[22] Munn D.H., Mellor A.L. (2004). IDO and tolerance to tumors. Trends Mol. Med..

[23] Dürr S., Kindler V. (2013). Implication of indolamine 2,3 dioxygenase in the tolerance toward fetuses, tumors, and allografts. J. Leukoc. Biol..

[24] Mazarei G., Leavitt B.R. (2015). Indoleamine 2,3 dioxygenase as a potential therapeutic target in Huntington’s disease. J. Huntingt. Dis..

[25] Widner B., Leblhuber F., Fuchs D. (2002). Increased neopterin production and tryptophan degradation in advanced Parkinson’s disease. J. Neural Transm..

[26] Mellor A.L., Baban B., Chandler P., Marshall B., Jhaver K., Hansen A., Koni P.A., Iwashima M., Munn D.H. (2003). Cutting edge: Induced indoleamine 2, 3 dioxygenase expression in dendritic cell subsets suppresses T cell clonal expansion. J. Immunol..

[27] Bessede A., Gargaro M., Pallotta M.T., Matino D., Servillo G., Brunacci C., Bicciato S., Mazza E.M., Macchiarulo A., Vacca C. (2014). Aryl hydrocarbon receptor control of a disease tolerance defence pathway. Nature.

[28] Guillemin G.J., Smith D.G., Smythe G.A., Armati P.J., Brew B.J. (2003). Expression of the kynurenine pathway enzymes in human microglia and macrophages. Adv. Exp. Med. Biol..

[29] Jones S.P., Franco N.F., Varney B., Sundaram G., Brown D.A., De Bie J., Lim C.K., Guillemin G.J., Brew B.J. (2015). Expression of the kynurenine pathway in human peripheral blood mononuclear cells: Implications for inflammatory and neurodegenerative disease. PLoS ONE.

[30] Guillemin G.J., Cullen K.M., Lim C.K., Smythe G.A., Garner B., Kapoor V., Takikawa O., Brew B.J. (2007). Characterization of the kynurenine pathway in human neurons. J. Neurosci..

[31] Vécsei L., Szalárdy L., Fülöp F., Toldi J. (2013). Kynurenines in the CNS: Recent advances and new questions. Nat. Rev. Drug Discov..

[32] Guillemin G.J., Kerr S.J., Brew B.J. (2005). Involvement of quinolinic acid in AIDS dementia complex. Neurotox. Res..

[33] Pierozan P., Biasibetti H., Schmitz F., Ávila H., Parisi M.M., Barbe-Tuana F., Wyse A.T.S., Pessoa-Pureur R. (2016). Quinolinic acid neurotoxicity: Differential roles of astrocytes and microglia via FGF-2-mediated signaling in redox-linked cytoskeletal changes. Biochim. Biophys. Acta BBA Mol. Cell Res..

[34] Chiarugi A., Meli E., Moroni F. (2001). Similarities and differences in the neuronal death processes activated by 3OH-kynurenine and quinolinic acid. J. Neurochem..

[35] Ramírez-Ortega D., Ramiro-Salazar A., González-Esquivel D., Ríos C., Pineda B., Pérez de la Cruz V. (2017). 3-Hydroxykynurenine and 3-hydroxyanthranilic acid enhance the toxicity induced by copper in rat astrocyte culture. Oxidative Med. Cell. Longev..

[36] Goldstein L.E., Leopold M.C., Huang X., Atwood C.S., Saunders A.J., Hartshorn M., Lim J.T., Faget K.Y., Muffat J.A., Scarpa R.C. (2000). 3-Hydroxykynurenine and 3-hydroxyanthranilic acid generate hydrogen peroxide and promote α-crystallin cross-linking by metal ion reduction. Biochemistry.

[37] Grant R., Coggan S., Smythe G. (2009). The physiological action of picolinic acid in the human brain. Int. J. Tryptophan Res..

[38] La Cruz V.P.-D., Carrillo-Mora P., Santamaría A. (2012). Quinolinic acid, an endogenous molecule combining excitotoxicity, oxidative stress and other toxic mechanisms. Int. J. Tryptophan Res..

[39] Rafice S.A., Chauhan N., Efimov I., Basran J., Raven E.L. (2009). Oxidation of L-tryptophan in biology: A comparison between tryptophan 2, 3-dioxygenase and indoleamine 2, 3-dioxygenase. Biochem. Soc. Trans..

[40] Meng B., Wu D., Gu J., Ouyang S., Ding W., Liu Z.J. (2014). Structural and functional analyses of human tryptophan 2, 3-dioxygenase. Proteins Struct. Funct. Bioinform..

[41] Ren S., Correia M.A. (2000). Heme: A regulator of rat hepatic tryptophan 2,3-dioxygenase?. Arch. Biochem. Biophys..

[42] Basile M.S., Mazzon E., Fagone P., Longo A., Russo A., Fallico M., Bonfiglio V., Nicoletti F., Avitabile T., Reibaldi M. (2019). Immunobiology of uveal melanoma: State of the art and therapeutic targets. Front. Oncol..

[43] Li J.S., Han Q., Fang J., Rizzi M., James A.A., Li J. (2007). Biochemical mechanisms leading to tryptophan 2,3-dioxygenase activation. Arch. Insect Biochem. Physiol..

[44] Maddison D.C., Giorgini F. (2015). The kynurenine pathway and neurodegenerative disease. Semin. Cell Dev. Biol..

[45] Konan K.V., Taylor M.W. (1996). Importance of the two interferon-stimulated response element (ISRE) sequences in the regulation of the human indoleamine 2,3-dioxygenase gene. J. Biol. Chem..

[46] Campbell B.M., Charych E., Lee A.W., Möller T. (2014). Kynurenines in CNS disease: Regulation by inflammatory cytokines. Front. Neurosci..

[47] Takikawa O., Kuroiwa T., Yamazaki F., Kido R. (1988). Mechanism of interferon-gamma action. Characterization of indoleamine 2,3-dioxygenase in cultured human cells induced by interferon-gamma and evaluation of the enzyme-mediated tryptophan degradation in its anticellular activity. J. Biol. Chem..

[48] Zunszain P.A., Anacker C., Cattaneo A., Choudhury S., Musaelyan K., Myint A.M., Thuret S., Price J., Pariante C.M. (2012). Interleukin-1β: A new regulator of the kynurenine pathway affecting human hippocampal neurogenesis. Neuropsychopharmacology.

[49] Connor T.J., Starr N., O’Sullivan J.B., Harkin A. (2008). Induction of indolamine 2,3-dioxygenase and kynurenine 3-monooxygenase in rat brain following a systemic inflammatory challenge: A role for IFN-γ?. Neurosci. Lett..

[50] Molteni R., Macchi F., Zecchillo C., Dell’Agli M., Colombo E., Calabrese F., Guidotti G., Racagni G., Riva M.A. (2013). Modulation of the inflammatory response in rats chronically treated with the antidepressant agomelatine. Eur. Neuropsychopharmacol..

[51] Giorgini F., Möller T., Kwan W., Zwilling D., Wacker J.L., Hong S., Tsai L.C., Cheah C.S., Schwarcz R., Guidetti P. (2008). Histone deacetylase inhibition modulates kynurenine pathway activation in yeast, microglia, and mice expressing a mutant huntingtin fragment. J. Biol. Chem..

[52] Kowalska M., Fijałkowski Ł., Nowaczyk A. (2021). Assessment of Paroxetine Molecular Interactions with Selected Monoamine and γ-Aminobutyric Acid Transporters. Int. J. Mol. Sci..

[53] Zhang S., Collier M.E.W., Heyes D.J., Giorgini F., Scrutton N.S. (2021). Advantages of brain penetrating inhibitors of kynurenine-3-monooxygenase for treatment of neurodegenerative diseases. Arch. Biochem. Biophys..

[54] Dang Y., Dale W.E., Brown O.R. (2000). Comparative effects of oxygen on indoleamine 2,3-dioxygenase and tryptophan 2,3-dioxygenase of the kynurenine pathway. Free Radic. Biol. Med..

[55] Gál E.M., Sherman A.D. (1980). L-kynurenine: Its synthesis and possible regulatory function in brain. Neurochem. Res..

[56] Speciale C., Schwarcz R. (1990). Uptake of kynurenine into rat brain slices. J. Neurochem..

[57] Guillemin G.J., Kerr S.J., Smythe G.A., Smith D.G., Kapoor V., Armati P.J., Croitoru J., Brew B.J. (2001). Kynurenine pathway metabolism in human astrocytes: A paradox for neuronal protection. J. Neurochem..

[58] Pocivavsek A., Notarangelo F.M., Wu H.-Q., Bruno J.P., Schwarcz R. (2016). Astrocytes as pharmacological targets in the treatment of schizophrenia: Focus on kynurenic acid. Handbook of Behavioral Neuroscience.

[59] Gramsbergen J.B., Hodgkins P.S., Rassoulpour A., Turski W.A., Guidetti P., Schwarcz R. (1997). Brain-specific modulation of kynurenic acid synthesis in the rat. J. Neurochem..

[60] Rassoulpour A., Wu H.Q., Poeggeler B., Schwarcz R. (1998). Systemic d-amphetamine administration causes a reduction of kynurenic acid levels in rat brain. Brain Res..

[61] Speciale C., Schwarcz R. (1993). On the production and disposition of quinolinic acid in rat brain and liver slices. J. Neurochem..

[62] Heyes M.P., Saito K., Major E.O., Milstien S., Markey S.P., Vickers J.H. (1993). A mechanism of quinolinic acid formation by brain in inflammatory neurological disease. Attenuation of synthesis from L-tryptophan by 6-chlorotryptophan and 4-chloro-3-hydroxyanthranilate. Brain.

[63] Foster A.C., White R.J., Schwarcz R. (1986). Synthesis of quinolinic acid by 3-hydroxyanthranilic acid oxygenase in rat brain tissue in vitro. J. Neurochem..

[64] Fukui S., Schwarcz R., Rapoport S.I., Takada Y., Smith Q.R. (1991). Blood-brain barrier transport of kynurenines: Implications for brain synthesis and metabolism. J. Neurochem..

[65] Cardinale A., Calabrese V., de Iure A., Picconi B. (2021). Alpha-Synuclein as a Prominent Actor in the Inflammatory Synaptopathy of Parkinson’s Disease. Int. J. Mol. Sci..

[66] Guidetti P., Schwarcz R. (1999). 3-Hydroxykynurenine potentiates quinolinate but not NMDA toxicity in the rat striatum. Eur. J. Neurosci..

[67] Jovanovic F., Candido K.D., Knezevic N.N. (2020). The Role of the Kynurenine Signaling Pathway in Different Chronic Pain Conditions and Potential Use of Therapeutic Agents. Int. J. Mol. Sci..

[68] Rojewska E., Ciapała K., Piotrowska A., Makuch W., Mika J. (2018). Pharmacological inhibition of indoleamine 2, 3-dioxygenase-2 and kynurenine 3-monooxygenase, enzymes of the kynurenine pathway, significantly diminishes neuropathic pain in a rat model. Front. Pharmacol..

[69] Török N., Maszlag-Török R., Molnár K., Szolnoki Z., Somogyvári F., Boda K., Tanaka M., Klivényi P., Vécsei L. (2020). Single Nucleotide Polymorphisms of Indoleamine 2, 3-Dioxygenase 1 Influenced the Age Onset of Parkinson’s Disease. Preprint.

[70] Jones S.P., Guillemin G.J., Brew B.J. (2013). The kynurenine pathway in stem cell biology. Int. J. Tryptophan Res..

[71] Lugo-Huitrón R., Ugalde Muñiz P., Pineda B., Pedraza-Chaverrí J., Ríos C., Pérez-de la Cruz V. (2013). Quinolinic acid: An endogenous neurotoxin with multiple targets. Oxidative Med. Cell. Longev..

[72] Perkins M.N., Stone T.W. (1983). Pharmacology and regional variations of quinolinic acid-evoked excitations in the rat central nervous system. J. Pharm. Exp..

[73] Vandresen-Filho S., Martins W.C., Bertoldo D.B., Mancini G., De Bem A.F., Tasca C.I. (2015). Cerebral cortex, hippocampus, striatum and cerebellum show differential susceptibility to quinolinic acid-induced oxidative stress. Neurol. Sci..

[74] Kumar U. (2004). Characterization of striatal cultures with the effect of QUIN and NMDA. Neurosci. Res..

[75] Chen Y., Brew B.J., Guillemin G.J. (2011). Characterization of the kynurenine pathway in NSC-34 cell line: Implications for amyotrophic lateral sclerosis. J. Neurochem..

[76] Ting K.K., Brew B.J., Guillemin G.J. (2009). Effect of quinolinic acid on human astrocytes morphology and functions: Implications in Alzheimer’s disease. J. Neuroinflamm..

[77] Santamaría A., Galván-Arzate S., Lisý V., Ali S.F., Duhart H.M., Osorio-Rico L., Ríos C., St’astný F. (2001). Quinolinic acid induces oxidative stress in rat brain synaptosomes. Neuroreport.

[78] Santamaría A., Jiménez-Capdeville M.E., Camacho A., Rodríguez-Martínez E., Flores A., Galván-Arzate S. (2001). In vivo hydroxyl radical formation after quinolinic acid infusion into rat corpus striatum. Neuroreport.

[79] Pláteník J., Stopka P., Vejrazka M., Stípek S. (2001). Quinolinic acid-iron(ii) complexes: Slow autoxidation, but enhanced hydroxyl radical production in the Fenton reaction. Free Radic. Res..

[80] Steiner J., Bogerts B., Sarnyai Z., Walter M., Gos T., Bernstein H.G., Myint A.M. (2012). Bridging the gap between the immune and glutamate hypotheses of schizophrenia and major depression: Potential role of glial NMDA receptor modulators and impaired blood-brain barrier integrity. World J. Biol. Psychiatry.

[81] St’astný F., Skultétyová I., Pliss L., Jezová D. (2000). Quinolinic acid enhances permeability of rat brain microvessels to plasma albumin. Brain Res. Bull..

[82] Pierozan P., Zamoner A., Soska A.K., Silvestrin R.B., Loureiro S.O., Heimfarth L., Mello e Souza T., Wajner M., Pessoa-Pureur R. (2010). Acute intrastriatal administration of quinolinic acid provokes hyperphosphorylation of cytoskeletal intermediate filament proteins in astrocytes and neurons of rats. Exp. Neurol..

[83] Rahman A., Ting K., Cullen K.M., Braidy N., Brew B.J., Guillemin G.J. (2009). The excitotoxin quinolinic acid induces tau phosphorylation in human neurons. PLoS ONE.

[84] Lee M.-C., Ting K.K., Adams S., Brew B.J., Chung R., Guillemin G.J. (2010). Characterisation of the Expression of NMDA Receptors in Human Astrocytes. PLoS ONE.

[85] Sundaram G., Brew B.J., Jones S.P., Adams S., Lim C.K., Guillemin G.J. (2014). Quinolinic acid toxicity on oligodendroglial cells: Relevance for multiple sclerosis and therapeutic strategies. J. Neuroinflamm..

[86] Guillemin G.J. (2012). Quinolinic acid, the inescapable neurotoxin. FEBS J..

[87] Guillemin G.J., Williams K.R., Smith D.G., Smythe G.A., Croitoru-Lamoury J., Brew B.J., Allegri G., Costa C.V.L., Ragazzi E., Steinhart H., Varesio L. (2003). Quinolinic Acid In the Pathogenesis of Alzheimer’s Disease. Developments in Tryptophan and Serotonin Metabolism.

[88] Montgomery E.B., He H. (2016). Deep Brain Stimulation Frequency—A Divining Rod for New and Novel Concepts of Nervous System Function and Therapy. Brain Sci..

[89] Kepplinger B., Baran H., Sedlnitzky-Semler B., Badawi N.-R., Erhart H. (2011). Stochastic Resonance Activity Influences Serum Tryptophan Metabolism in Healthy Human Subjects. Int. J. Tryptophan Res..

[90] Ji F., Wei J., Luan H., Li M., Cai Z. (2019). Study of metabolic disorders associated with BDE-47 exposure in Drosophila model by MS-based metabolomics. Ecotoxicol. Environ. Saf..

[91] Ogawa T., Matson W.R., Beal M.F., Myers R.H., Bird E.D., Milbury P., Saso S. (1992). Kynurenine pathway abnormalities in Parkinson’s disease. Neurology.

[92] Beal M.F., Matson W.R., Storey E., Milbury P., Ryan E.A., Ogawa T., Bird E.D. (1992). Kynurenic acid concentrations are reduced in Huntington’s disease cerebral cortex. J. Neurol. Sci..

[93] Zinger A., Barcia C., Herrero M.T., Guillemin G.J. (2011). The involvement of neuroinflammation and kynurenine pathway in Parkinson’s disease. Parkinsons Dis..

[94] Widner B., Laich A., Sperner-Unterweger B., Ledochowski M., Fuchs D. (2002). Neopterin production, tryptophan degradation, and mental depression—What is the link?. Brain Behav. Immun..

[95] Hartai Z., Klivenyi P., Janaky T., Penke B., Dux L., Vecsei L. (2005). Kynurenine metabolism in plasma and in red blood cells in Parkinson’s disease. J. Neurol. Sci..

[96] Barth M.C., Ahluwalia N., Anderson T.J.T., Hardy G.J., Sinha S., Alvarez-Cardona J.A., Pruitt I.E., Rhee E.P., Colvin R.A., Gerszten R.E. (2009). Kynurenic acid triggers firm arrest of leukocytes to vascular endothelium under flow conditions. J. Biol. Chem..

[97] Fujigaki S., Saito K., Sekikawa K., Tone S., Takikawa O., Fujii H., Wada H., Noma A., Seishima M. (2001). Lipopolysaccharide induction of indoleamine 2,3-dioxygenase is mediated dominantly by an IFN-gamma-independent mechanism. Eur. J. Immunol..

[98] Pinto J.T., Krasnikov B.F., Alcutt S., Jones M.E., Dorai T., Villar M.T., Artigues A., Li J., Cooper A.J.L. (2014). Kynurenine aminotransferase III and glutamine transaminase L are identical enzymes that have cysteine S-conjugate β-lyase activity and can transaminate L-selenomethionine. J. Biol. Chem..

[99] McNally L., Bhagwagar Z., Hannestad J. (2008). Inflammation, glutamate, and glia in depression: A literature review. CNS Spectr..

[100] Marin C., Jimenez A., Bonastre M., Chase T.N., Tolosa E. (2000). Non-NMDA receptor-mediated mechanisms are involved in levodopa-induced motor response alterations in Parkinsonian rats. Synapse.

[101] Ceresoli-Borroni G., Guidetti P., Amori L., Pellicciari R., Schwarcz R. (2007). Perinatal kynurenine 3-hydroxylase inhibition in rodents: Pathophysiological implications. J. Neurosci. Res..

[102] Grégoire L., Rassoulpour A., Guidetti P., Samadi P., Bédard P.J., Izzo E., Schwarcz R., Di Paolo T. (2008). Prolonged kynurenine 3-hydroxylase inhibition reduces development of levodopa-induced dyskinesias in parkinsonian monkeys. Behav. Brain Res..

[103] Samadi P., Grégoire L., Rassoulpour A., Guidetti P., Izzo E., Schwarcz R., Bédard P.J. (2005). Effect of kynurenine 3-hydroxylase inhibition on the dyskinetic and antiparkinsonian responses to levodopa in parkinsonian monkeys. Mov. Disord..

[104] Abdel-Daim M.M., Abo-El-Sooud K., Aleya L., Bungău S.G., Najda A., Saluja R. (2018). Alleviation of Drugs and Chemicals Toxicity: Biomedical Value of Antioxidants. Oxidative Med. Cell. Longev..

[105] Wonodi I., Stine O.C., Sathyasaikumar K.V., Roberts R.C., Mitchell B.D., Hong L.E., Kajii Y., Thaker G.K., Schwarcz R. (2011). Downregulated kynurenine 3-monooxygenase gene expression and enzyme activity in schizophrenia and genetic association with schizophrenia endophenotypes. Arch. Gen. Psychiatry.

[106] Behl T., Kaur G., Fratila O., Buhas C., Judea-Pusta C.T., Negrut N., Bustea C., Bungau S. (2021). Cross-talks among GBA Gene Mutations, GCase, and α-synuclein in GBA Associated Parkinson’s Disease with their Targeted Therapeutic Approaches: A Comprehensive Review. Transl. Neurodegener..

[107] Miranda A.F., Boegman R.J., Beninger R.J., Jhamandas K. (1997). Protection against quinolinic acid-mediated excitotoxicity in nigrostriatal dopaminergic neurons by endogenous kynurenic acid. Neuroscience.

[108] Wu H.Q., Rassoulpour A., Schwarcz R. (2002). Effect of systemic L-DOPA administration on extracellular kynurenate levels in the rat striatum. J. Neural Transm..

[109] Brotchie J.M., Mitchell I.J., Sambrook M.A., Crossman A.R. (1991). Alleviation of parkinsonism by antagonism of excitatory amino acid transmission in the medial segment of the globus pallidus in rat and primate. Mov. Disord..

[110] Silva-Adaya D., Pérez-De La Cruz V., Villeda-Hernández J., Carrillo-Mora P., González-Herrera I.G., García E., Colín-Barenque L., Pedraza-Chaverrí J., Santamaría A. (2011). Protective effect of L-kynurenine and probenecid on 6-hydroxydopamine-induced striatal toxicity in rats: Implications of modulating kynurenate as a protective strategy. Neurotoxicol. Teratol..

[111] Wu H.Q., Rassoulpour A., Schwarcz R. (2007). Kynurenic acid leads, dopamine follows: A new case of volume transmission in the brain?. J. Neural. Transm..

[112] Oxenkrug G., van der Hart M., Roeser J., Summergrad P. (2017). Peripheral Tryptophan—Kynurenine Metabolism Associated with Metabolic Syndrome is Different in Parkinson’s and Alzheimer’s Diseases. Endocrinol. Diabetes. Metab. J..

[113] Tanaka M., Vécsei L. (2020). Monitoring the redox status in multiple sclerosis. Biomedicines.

[114] Di Meo S., Reed T.T., Venditti P., Victor V.M. (2016). Role of ROS and RNS sources in physiological and pathological conditions. Oxidative Med. Cell. Longev..

[115] Cores Á., Piquero M., Villacampa M., León R., Menéndez J.C. (2020). NRF2 regulation processes as a source of potential drug targets against neurodegenerative diseases. Biomolecules.

[116] Vargas-Mendoza N., Morales-González Á., Madrigal-Santillán E.O., Madrigal-Bujaidar E., Álvarez-González I., García-Melo L.F., Anguiano-Robledo L., Fregoso-Aguilar T., Morales-Gonzalez J.A. (2019). Antioxidant and adaptative response mediated by Nrf2 during physical exercise. Antioxidants.

[117] Di Rosa G., Brunetti G., Scuto M., Trovato Salinaro A., Calabrese E.J., Crea R., Schmitz-Linneweber C., Calabrese V., Saul N. (2020). Healthspan enhancement by olive polyphenols in C. elegans wild type and Parkinson’s models. Int. J. Mol. Sci..

[118] Carrillo-Mora P., Pérez-De la Cruz V., Estrada-Cortés B., Toussaint-González P., Martínez-Cortéz J.A., Rodríguez-Barragán M., Quinzaños-Fresnedo J., Rangel-Caballero F., Gamboa-Coria G., Sánchez-Vázquez I. (2020). Serum Kynurenines Correlate With Depressive Symptoms and Disability in Poststroke Patients: A Cross-sectional Study. Neurorehabil. Neural Repair.

[119] Vazquez S., Garner B., Sheil M.M., Truscott R.J.W. (2000). Characterisation of the major autoxidation products of 3-hydroxykynurenine under physiological conditions. Free Radic. Res..

[120] Anderson G., Maes M. (2014). Neurodegeneration in Parkinson’s disease: Interactions of oxidative stress, tryptophan catabolites and depression with mitochondria and sirtuins. Mol. Neurobiol..

[121] Anderson G., Maes M. (2014). TRYCAT pathways link peripheral inflammation, nicotine, somatization and depression in the etiology and course of Parkinson’s disease. CNS Neurol. Disord. Drug Targets.

[122] Capuron L., Miller A.H. (2011). Immune system to brain signaling: Neuropsychopharmacological implications. Pharmacol. Ther..

[123] Erabi H., Okada G., Shibasaki C., Setoyama D., Kang D., Takamura M., Yoshino A., Fuchikami M., Kurata A., Kato T.A. (2020). Kynurenic acid is a potential overlapped biomarker between diagnosis and treatment response for depression from metabolome analysis. Sci. Rep..

[124] Hunt B.S.C., Cordeiro T.M., Robert S., de Dios C., Leal V.A.C., Soares J.C., Robert D., Antonio T., Sudhakar S.M. (2020). Effect of mmune Activation on the Kynurenine Pathway and Depression Symptoms–A Systematic Review and Meta-Analysis. Neurosci. Biobehav. Rev..

[125] Bay-Richter C., Linderholm K.R., Lim C.K., Samuelsson M., Träskman-Bendz L., Guillemin G.J., Erhardt S., Brundin L. (2015). A role for inflammatory metabolites as modulators of the glutamate N-methyl-D-aspartate receptor in depression and suicidality. Brain Behav. Immun..

[126] Savitz J., Drevets W.C., Smith C.M., Victor T.A., Wurfel B.E., Bellgowan P.S., Bodurka J., Teague T.K., Dantzer R. (2015). Putative neuroprotective and neurotoxic kynurenine pathway metabolites are associated with hippocampal and amygdalar volumes in subjects with major depressive disorder. Neuropsychopharmacology.

[127] Lindqvist D., Kaufman E., Brundin L., Hall S., Surova Y., Hansson O. (2012). Non-motor symptoms in patients with Parkinson’s disease-correlations with inflammatory cytokines in serum. PLoS ONE.

[128] Khan M.M., Kempuraj D., Thangavel R., Zaheer A. (2013). Protection of MPTP-induced neuroinflammation and neurodegeneration by Pycnogenol. Neurochem. Int..

[129] Tas S.W., Vervoordeldonk M.J., Hajji N., Schuitemaker J.H.N., van der Sluijs K.F., May M.J., Ghosh S., Kapsenberg M.L., Tak P.P., de Jong E.C. (2007). Noncanonical NF-κB signaling in dendritic cells is required for indoleamine 2,3-dioxygenase (IDO) induction and immune regulation. Blood.

[130] Steiner J., Walter M., Gos T., Guillemin G.J., Bernstein H.-G., Sarnyai Z., Mawrin C., Brisch R., Bielau H., zu Schwabedissen L.M. (2011). Severe depression is associated with increased microglial quinolinic acid in subregions of the anterior cingulate gyrus: Evidence for an immune-modulated glutamatergic neurotransmission?. J. Neuroinflamm..

[131] De Carvalho L.P., Bochet P., Rossier J. (1996). The endogenous agonist quinolinic acid and the non endogenous homoquinolinic acid discriminate between NMDAR2 receptor subunits. Neurochem. Int..

[132] Beal M.F., Kowall N.W., Ellison D.W., Mazurek M.F., Swartz K.J., Martin J.B. (1986). Replication of the neurochemical characteristics of Huntington’s disease by quinolinic acid. Nature.

[133] Foster A.C., Collins J.F., Schwarcz R. (1983). On the excitotoxic properties of quinolinic acid, 2,3-piperidine dicarboxylic acids and structurally related compounds. Neuropharmacology.

[134] Shear D.A., Dong J., Haik-Creguer K.L., Bazzett T.J., Albin R.L., Dunbar G.L. (1998). Chronic Administration of Quinolinic Acid in the Rat Striatum Causes Spatial Learning Deficits in a Radial Arm Water Maze Task. Exp. Neurol..

[135] Vazey E.M., Chen K., Hughes S.M., Connor B. (2006). Transplanted adult neural progenitor cells survive, differentiate and reduce motor function impairment in a rodent model of Huntington’s disease. Exp. Neurol..

[136] McGeer E.G., Singh E. (1984). Neurotoxic effects of endogenous materials: Quinolinic acid, l-pyroglutamic acid, and thyroid releasing hormone (TRH). Exp. Neurol..

[137] Perkins M.N., Stone T.W. (1982). An iontophoretic investigation of the actions of convulsant kynurenines and their interaction with the endogenous excitant quinolinic acid. Brain Res..

[138] Lee D.Y., Lee K.S., Lee H.J., Noh Y.H., Kim D.H., Lee J.Y., Cho S.H., Yoon O.J., Lee W.B., Kim K.Y. (2008). Kynurenic acid attenuates MPP(+)-induced dopaminergic neuronal cell death via a Bax-mediated mitochondrial pathway. Eur. J. Cell Biol..

[139] Butler E.G., Bourke D.W., Finkelstein D.I., Horne M.K. (1997). The effects of reversible inactivation of the subthalamo-pallidal pathway on the behaviour of naive and hemiparkinsonian monkeys. J. Clin. Neurosci..

[140] Zádori D., Klivényi P., Plangár I., Toldi J., Vécsei L. (2011). Endogenous neuroprotection in chronic neurodegenerative disorders: With particular regard to the kynurenines. J. Cell. Mol. Med..

[141] Johnson K.A., Conn P.J., Niswender C.M. (2009). Glutamate receptors as therapeutic targets for Parkinson’s disease. CNS Neurol. Disord. Drug Targets.

[142] Kubicova L., Hadacek F., Bachmann G., Weckwerth W., Chobot V. (2019). Coordination Complex Formation and Redox Properties of Kynurenic and Xanthurenic Acid Can Affect Brain Tissue Homeodynamics. Antioxidants.

[143] Del Tredici K., Braak H. (2008). A not entirely benign procedure: Progression of Parkinson’s disease. Acta Neuropathol..

[144] Mulak A., Bonaz B. (2015). Brain-gut-microbiota axis in Parkinson’s disease. World J. Gastroenterol..

[145] Böttner M., Zorenkov D., Hellwig I., Barrenschee M., Harde J., Fricke T., Deuschl G., Egberts J.H., Becker T., Fritscher-Ravens A. (2012). Expression pattern and localization of alpha-synuclein in the human enteric nervous system. Neurobiol. Dis..

[146] Forsyth C.B., Shannon K.M., Kordower J.H., Voigt R.M., Shaikh M., Jaglin J.A., Estes J.D., Dodiya H.B., Keshavarzian A. (2011). Increased Intestinal Permeability Correlates with Sigmoid Mucosa alpha-Synuclein Staining and Endotoxin Exposure Markers in Early Parkinson’s Disease. PLoS ONE.

[147] Devos D., Lebouvier T., Lardeux B., Biraud M., Rouaud T., Pouclet H., Coron E., Bruley des Varannes S., Naveilhan P., Nguyen J.M. (2013). Colonic inflammation in Parkinson’s disease. Neurobiol. Dis..

[148] Westfall S., Lomis N., Kahouli I., Dia S.Y., Singh S.P., Prakash S. (2017). Microbiome, probiotics and neurodegenerative diseases: Deciphering the gut brain axis. Cell Mol. Life Sci..

[149] Dehhaghi M., Kazemi Shariat Panahi H., Guillemin G.J. (2019). Microorganisms, Tryptophan Metabolism, and Kynurenine Pathway: A Complex Interconnected Loop Influencing Human Health Status. Int. J. Tryptophan Res..

[150] Gao J., Xu K., Liu H., Liu G., Bai M., Peng C., Li T., Yin Y. (2018). Impact of the Gut Microbiota on Intestinal Immunity Mediated by Tryptophan Metabolism. Front. Cell. Infect. Microbiol..

[151] Vujkovic-Cvijin I., Dunham R.M., Iwai S., Maher M.C., Albright R.G., Broadhurst M.J., Hernandez R.D., Lederman M.M., Huang Y., Somsouk M. (2013). Dysbiosis of the gut microbiota is associated with HIV disease progression and tryptophan catabolism. Sci. Transl. Med..

[152] Makkar R., Behl T., Bungau S., Zengin G., Mehta V., Kumar A., Uddin M.S., Ashraf G.M., Abdel-Daim M.M., Arora S. (2020). Nutraceuticals in Neurological Disorders. Int. J. Mol. Sci..

[153] O’Farrell K., Harkin A. (2017). Stress-related regulation of the kynurenine pathway: Relevance to neuropsychiatric and degenerative disorders. Neuropharmacology.

[154] Shoaie S., Ghaffari P., Kovatcheva-Datchary P., Mardinoglu A., Sen P., Pujos-Guillot E., de Wouters T., Juste C., Rizkalla S., Chilloux J. (2015). Quantifying Diet-Induced Metabolic Changes of the Human Gut Microbiome. Cell Metab..

[155] El Aidy S., Dinan T., Cryan J. (2014). Immune modulation of the brain-gut-microbe axis. Front. Microbiol..

[156] Clarke S.F., Murphy E.F., O’Sullivan O., Ross R.P., O’Toole P.W., Shanahan F., Cotter P.D. (2013). Targeting the Microbiota to Address Diet-Induced Obesity: A Time Dependent Challenge. PLoS ONE.

[157] Wikoff W.R., Anfora A.T., Liu J., Schultz P.G., Lesley S.A., Peters E.C., Siuzdak G. (2009). Metabolomics analysis reveals large effects of gut microflora on mammalian blood metabolites. Proc. Natl. Acad. Sci. USA.

[158] Tavassoly O., Sade D., Bera S., Shaham-Niv S., Vocadlo D.J., Gazit E. (2018). Quinolinic Acid Amyloid-like Fibrillar Assemblies Seed α-Synuclein Aggregation. J. Mol. Biol..

[159] Lewitt P.A., Li J., Lu M., Beach T.G., Adler C.H., Guo L. (2013). 3-hydroxykynurenine and other Parkinson’s disease biomarkers discovered by metabolomic analysis. Mov. Disord..

[160] Vilas D., Fernández-Santiago R., Sanchez E., Azcona L.J., Santos-Montes M., Casquero P., Argandoña L., Tolosa E., Paisán-Ruiz C. (2017). A Novel p.Glu298Lys Mutation in the ACMSD Gene in Sporadic Parkinson’s Disease. J. Parkinsons Dis..

[161] Chahine L.M., Stern M.B., Chen-Plotkin A. (2014). Blood-based biomarkers for Parkinson’s disease. Parkinsonism Relat. Disord..

[162] Baković J., López Martínez D., Nikolaou S., Yu B.Y.K., Tossounian M.-A., Tsuchiya Y., Thrasivoulou C., Filonenko V., Gout I. (2021). Regulation of the CoA Biosynthetic Complex Assembly in Mammalian Cells. Int. J. Mol. Sci..

[163] Sas K., Szabó E., Vécsei L. (2018). Mitochondria, Oxidative Stress and the Kynurenine System, with a Focus on Ageing and Neuroprotection. Molecules.

[164] Burgos K., Malenica I., Metpally R., Courtright A., Rakela B., Beach T., Shill H., Adler C., Sabbagh M., Villa S. (2014). Profiles of Extracellular miRNA in Cerebrospinal Fluid and Serum from Patients with Alzheimer’s and Parkinson’s Diseases Correlate with Disease Status and Features of Pathology. PLoS ONE.

[165] Luan H., Liu L.-F., Meng N., Tang Z., Chua K.-K., Chen L.-L., Song J.-X., Mok V.C.T., Xie L.-X., Li M. (2015). LC–MS-Based Urinary Metabolite Signatures in Idiopathic Parkinson’s Disease. J. Proteome Res..

[166] Luan H., Liu L.F., Tang Z., Zhang M., Chua K.K., Song J.X., Mok V.C., Li M., Cai Z. (2015). Comprehensive urinary metabolomic profiling and identification of potential noninvasive marker for idiopathic Parkinson’s disease. Sci. Rep..

[167] Iwaoka K., Otsuka C., Maeda T., Yamahara K., Kato K., Takahashi K., Takahashi K., Terayama Y. (2020). Impaired metabolism of kynurenine and its metabolites in CSF of Parkinson’s disease. Neurosci. Lett..

[168] Demeter I., Nagy K., Gellért L., Vécsei L., Fülöp F., Toldi J. (2012). A novel kynurenic acid analog (SZR104) inhibits pentylenetetrazole-induced epileptiform seizures. An electrophysiological study: Special issue related to kynurenine. J. Neural Transm..

[169] Gellért L., Fuzik J., Göblös A., Sárközi K., Marosi M., Kis Z., Farkas T., Szatmári I., Fülöp F., Vécsei L. (2011). Neuroprotection with a new kynurenic acid analog in the four-vessel occlusion model of ischemia. Eur. J. Pharm..

[170] Zádori D., Klivényi P., Toldi J., Fülöp F., Vécsei L. (2012). Kynurenines in Parkinson’s disease: Therapeutic perspectives. J. Neural Transm..

[171] Wu H.Q., Lee S.C., Schwarcz R. (2000). Systemic administration of 4-chlorokynurenine prevents quinolinate neurotoxicity in the rat hippocampus. Eur. J. Pharm..

[172] Moffett J.R., Els T., Espey M.G., Walter S.A., Streit W.J., Namboodiri M.A. (1997). Quinolinate immunoreactivity in experimental rat brain tumors is present in macrophages but not in astrocytes. Exp. Neurol..

[173] Fukuyama K., Tanahashi S., Hoshikawa M., Shinagawa R., Okada M. (2014). Zonisamide regulates basal ganglia transmission via astroglial kynurenine pathway. Neuropharmacology.

[174] Copeland C.S., Neale S.A., Salt T.E. (2013). Actions of Xanthurenic acid, a putative endogenous Group II metabotropic glutamate receptor agonist, on sensory transmission in the thalamus. Neuropharmacology.

[175] Fazio F., Lionetto L., Molinaro G., Bertrand H.O., Acher F., Ngomba R.T., Notartomaso S., Curini M., Rosati O., Scarselli P. (2012). Cinnabarinic acid, an endogenous metabolite of the kynurenine pathway, activates type 4 metabotropic glutamate receptors. Mol. Pharm..

[176] Nicoletti F., Bockaert J., Collingridge G.L., Conn P.J., Ferraguti F., Schoepp D.D., Wroblewski J.T., Pin J.P. (2011). Metabotropic glutamate receptors: From the workbench to the bedside. Neuropharmacology.

[177] Duty S. (2010). Therapeutic potential of targeting group III metabotropic glutamate receptors in the treatment of Parkinson’s disease. Br. J. Pharmacol..

[178] Hamann M., Sander S.E., Richter A. (2008). Effects of the kynurenine 3-hydroxylase inhibitor Ro 61-8048 after intrastriatal injections on the severity of dystonia in the dt sz mutant. Eur. J. Pharm..

[179] Ouattara B., Belkhir S., Morissette M., Dridi M., Samadi P., Grégoire L., Meltzer L.T., Di Paolo T. (2009). Implication of NMDA receptors in the antidyskinetic activity of cabergoline, CI-1041, and Ro 61-8048 in MPTP monkeys with levodopa-induced dyskinesias. J. Mol. Neurosci..

[180] Pomplun S., Wang Y., Kirschner A., Kozany C., Bracher A., Hausch F. (2015). Rational Design and Asymmetric Synthesis of Potent and Neurotrophic Ligands for FK506-Binding Proteins (FKBPs). Angew. Chem. Int. Ed..

[181] Van der Perren A., Macchi F., Toelen J., Carlon M.S., Maris M., de Loor H., Kuypers D.R., Gijsbers R., Van den Haute C., Debyser Z. (2015). FK506 reduces neuroinflammation and dopaminergic neurodegeneration in an α-synuclein-based rat model for Parkinson’s disease. Neurobiol. Aging.

[182] Auluck P.K., Chan H.E., Trojanowski J.Q., Lee V.M.-Y., Bonini N.M. (2002). Chaperone suppression of α-synuclein toxicity in a Drosophila model for Parkinson’s disease. Science.

[183] Jones D.R., Moussaud S., McLean P. (2014). Targeting heat shock proteins to modulate α-synuclein toxicity. Ther. Adv. Neurol. Disord..

[184] Nagel F., Falkenburger B.H., Tönges L., Kowsky S., Pöppelmeyer C., Schulz J.B., Bähr M., Dietz G.P.H. (2008). Tat-Hsp70 protects dopaminergic neurons in midbrain cultures and in the substantia nigra in models of Parkinson’s disease. J. Neurochem..

[185] Tanabe A., Yamamura Y., Kasahara J., Morigaki R., Kaji R., Goto S. (2014). A novel tyrosine kinase inhibitor AMN107 (nilotinib) normalizes striatal motor behaviors in a mouse model of Parkinson’s disease. Front. Cell. Neurosci..

[186] Nuzzo D. (2021). Role of Natural Antioxidants on Neuroprotection and Neuroinflammation. Antioxidants.

[187] Pizzino G., Irrera N., Cucinotta M., Pallio G., Mannino F., Arcoraci V., Squadrito F., Altavilla D., Bitto A. (2017). Oxidative stress: Harms and benefits for human health. Oxidative Med. Cell. Longev..

[188] Oh Y., Do H.T.T., Kim S., Kim Y.-M., Chin Y.-W., Cho J. (2021). Memory-Enhancing Effects of Mangosteen Pericarp Water Extract through Antioxidative Neuroprotection and Anti-Apoptotic Action. Antioxidants.

[189] Burgos C., Muñoz-Mingarro D., Navarro I., Martín-Cordero C., Acero N. (2020). Neuroprotective Potential of Verbascoside Isolated from Acanthus mollis L. Leaves through Its Enzymatic Inhibition and Free Radical Scavenging Ability. Antioxidants.

[190] Capatina L., Todirascu-Ciornea E., Napoli E.M., Ruberto G., Hritcu L., Dumitru G. (2020). Thymus vulgaris Essential Oil Protects Zebrafish against Cognitive Dysfunction by Regulating Cholinergic and Antioxidants Systems. Antioxidants.

[191] Moisa C., Lupitu A., Pop G., Chambre D.R., Copolovici L., Cioca G., Bungau S., Copolovici D.M. (2019). Variation of the Chemical Composition of Thymus Vulgaris Essential Oils by Phenological Stages. Rev. Chim..

[192] Ramsey T.L., Liu Q., Massey B.W., Brennan M.D. (2013). Genotypic variation in the SV2C gene impacts response to atypical antipsychotics the CATIE study. Schizophr. Res..

[193] Khaliq Z.M., Bean B.P. (2010). Pacemaking in dopaminergic ventral tegmental area neurons: Depolarizing drive from background and voltage-dependent sodium conductances. J. Neurosci..

[194] Mosharov E.V., Larsen K.E., Kanter E., Phillips K.A., Wilson K., Schmitz Y., Krantz D.E., Kobayashi K., Edwards R.H., Sulzer D. (2009). Interplay between cytosolic dopamine, calcium, and α-synuclein causes selective death of substantia nigra neurons. Neuron.

[195] Liu Y., Harding M., Dore J., Chen X. (2017). Cav1. 2, but not Cav1. 3, L-type calcium channel subtype mediates nicotine-induced conditioned place preference in mice. Prog. Neuro Psychopharmacol. Biol. Psychiatry.

[196] Wang X., Saegusa H., Huntula S., Tanabe T. (2019). Blockade of microglial Cav1. 2 Ca^2+^ channel exacerbates the symptoms in a Parkinson’s disease model. Sci. Rep..

[197] Kang S., Cooper G., Dunne S.F., Luan C.-H., Surmeier D.J., Silverman R.B. (2013). Antagonism of L-type Ca^2+^ channels CaV1. 3 and CaV1. 2 by 1, 4-dihydropyrimidines and 4H-pyrans as dihydropyridine mimics. Bioorg. Med. Chem..

[198] Liu Y., Harding M., Pittman A., Dore J., Striessnig J., Rajadhyaksha A., Chen X. (2014). Cav1.2 and Cav1.3 L-type calcium channels regulate dopaminergic firing activity in the mouse ventral tegmental area. J. Neurophysiol..

[199] Turrens J.F. (2003). Mitochondrial formation of reactive oxygen species. J. Physiol..

[200] Behl T., Kaur G., Sehgal A., Bhardwaj S., Singh S., Buhas C., Judea-Pusta C., Uivarosan D., Munteanu M.A., Bungau S. (2021). Multifaceted Role of Matrix Metalloproteinases in Neurodegenerative Diseases: Pathophysiological and Therapeutic Perspectives. Int. J. Mol. Sci..

[201] Elstner M., Morris C.M., Heim K., Bender A., Mehta D., Jaros E., Klopstock T., Meitinger T., Turnbull D.M., Prokisch H. (2011). Expression analysis of dopaminergic neurons in Parkinson’s disease and aging links transcriptional dysregulation of energy metabolism to cell death. Acta Neuropathol..

[202] Catanesi M., d’Angelo M., Tupone M.G., Benedetti E., Giordano A., Castelli V., Cimini A. (2020). MicroRNAs dysregulation and mitochondrial dysfunction in neurodegenerative diseases. Int. J. Mol. Sci..

[203] López-Gambero A.J., Rosell-Valle C., Medina-Vera D., Navarro J.A., Vargas A., Rivera P., Sanjuan C., de Fonseca F.R., Suárez J. (2021). A Negative Energy Balance Is Associated with Metabolic Dysfunctions in the Hypothalamus of a Humanized Preclinical Model of Alzheimer’s Disease, the 5XFAD Mouse. Int. J. Mol. Sci..

[204] McNaught K.S.P., Jenner P. (2001). Proteasomal function is impaired in substantia nigra in Parkinson’s disease. Neurosci. Lett..

[205] Kawahata I., Fukunaga K. (2020). Degradation of tyrosine hydroxylase by the ubiquitin-proteasome system in the pathogenesis of Parkinson’s disease and dopa-responsive dystonia. Int. J. Mol. Sci..

[206] Ceulemans A.-G., Zgavc T., Kooijman R., Hachimi-Idrissi S., Sarre S., Michotte Y. (2010). The dual role of the neuroinflammatory response after ischemic stroke: Modulatory effects of hypothermia. J. Neuroinflamm..

[207] Liu C.-Y., Wang X., Liu C., Zhang H.-L. (2019). Pharmacological Targeting of Microglial Activation: New Therapeutic Approach. Front. Cell. Neurosci..

